# Tin Oxide Based Nanomaterials and Their Application as Anodes in Lithium‐Ion Batteries and Beyond

**DOI:** 10.1002/cssc.201901487

**Published:** 2019-08-30

**Authors:** Florian Zoller, Daniel Böhm, Thomas Bein, Dina Fattakhova‐Rohlfing

**Affiliations:** ^1^ Department of Chemistry and Center for NanoScience (CeNS) Ludwig-Maximilians-Universität München (LMU Munich) Butenandtstrasse 5-13 (E) 81377 Munich Germany; ^2^ Institute of Energy and Climate Research (IEK-1), Materials Synthesis and Processing Forschungszentrum Jülich GmbH Wilhelm-Johnen-Strasse 52425 Jülich Germany; ^3^ Faculty of Engineering and Center for Nanointegration, Duisburg-Essen (CENIDE) Universität Duisburg-Essen (UDE) Lotharstraße 1 47057 Duisburg Germany

**Keywords:** doping, electrochemistry, lithium, nanoparticles, tin

## Abstract

Herein, recent progress in the field of tin oxide (SnO_2_)‐based nanosized and nanostructured materials as conversion and alloying/dealloying‐type anodes in lithium‐ion batteries and beyond (sodium‐ and potassium‐ion batteries) is briefly discussed. The first section addresses the importance of the initial SnO_2_ micro‐ and nanostructure on the conversion and alloying/dealloying reaction upon lithiation and its impact on the microstructure and cyclability of the anodes. A further section is dedicated to recent advances in the fabrication of diverse 0D to 3D nanostructures to overcome stability issues induced by large volume changes during cycling. Additionally, the role of doping on conductivity and synergistic effects of redox‐active and ‐inactive dopants on the reversible lithium‐storage capacity and rate capability are discussed. Furthermore, the synthesis and electrochemical properties of nanostructured SnO_2_/C composites are reviewed. The broad research spectrum of SnO_2_ anode materials is finally reflected in a brief overview of recent work published on Na‐ and K‐ion batteries.

## Graphical abstract

**A matter of size and structure**: Large volume changes during SnO_2_ conversion and alloying of an anode limit its stability upon cycling. A brief overview of how primary particle size, nanostructuring, and composite formation influence the electrode performance and stability in SnO_2_ anode‐based lithium‐ion batteries is provided and complemented by recent reports on corresponding sodium and potassium systems.

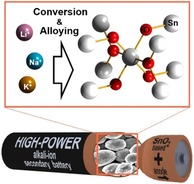

## 1 Introduction

Lithium‐ion batteries (LIBs) represent the most advanced electrochemical energy‐storage technology for powering mobile and consumer applications, with energy and power densities greatly exceeding those of other battery systems. Although enormous progress in the performance of LIBs has been achieved in recent decades, making even large‐scale energy storage applications, such as electric vehicles feasible, the constantly growing demand for electrical energy storage devices necessitates the development of novel battery chemistries to further increase the energy density on the cell level.^1^ By using materials with different energy‐storage mechanisms, such as alloying or conversion, instead of the state‐of‐the‐art insertion anode material, graphite, is a promising way to significantly increase the charge‐storage capacity.

Tin‐based conversion and alloying anode materials gained considerable attention in recent years due to their high theoretical capacity. Metallic tin, tin alloys, stannates, or tin chalcogenides such as tin (di)sulfide and tin (di)oxide were intensively investigated as battery anode materials.^2^ Among the listed materials classes, metallic tin features the highest theoretical capacity, but suffers from severe stability issues upon cycling. Although nanostructuring or alloying were shown to be promising concepts to improve long‐term stability, the use of metallic tin as an anode remains very challenging.^2^

Tin dioxide (SnO_2_) and layered sulfides (SnS or SnS_2_) exhibit comparable theoretical capacities. However, tin oxides show faster lithiation/delithiation kinetics and a greatly enhanced cyclability, whereas the Li insertion and conversion reaction with SnS_2_ is only partly reversible.^2^ Therefore, SnO_2_ is believed to be a potential candidate as an active anode material for next‐generation LIBs.

It was more than 20 years ago that tin oxide materials were reported, for the first time, by Idota et al. from the Fuji Photo Film Celltec Co. (Japan) company as highly promising anode materials.^3^ Since that time, tin oxide containing materials have gained tremendous attention due to the high theoretical and volumetric capacity, biological compatibility, environmental friendliness, and rather low cost. Moreover, the low discharge potential of SnO_2_ makes it even more attractive as an anode material in LIBs.^4^

The lithium reaction with SnO_2_ has been long believed to proceed through two major steps, namely, a conversion reaction followed by a subsequent alloying/dealloying process; this was substantiated by various in situ studies.^5^

**Figure 1:**
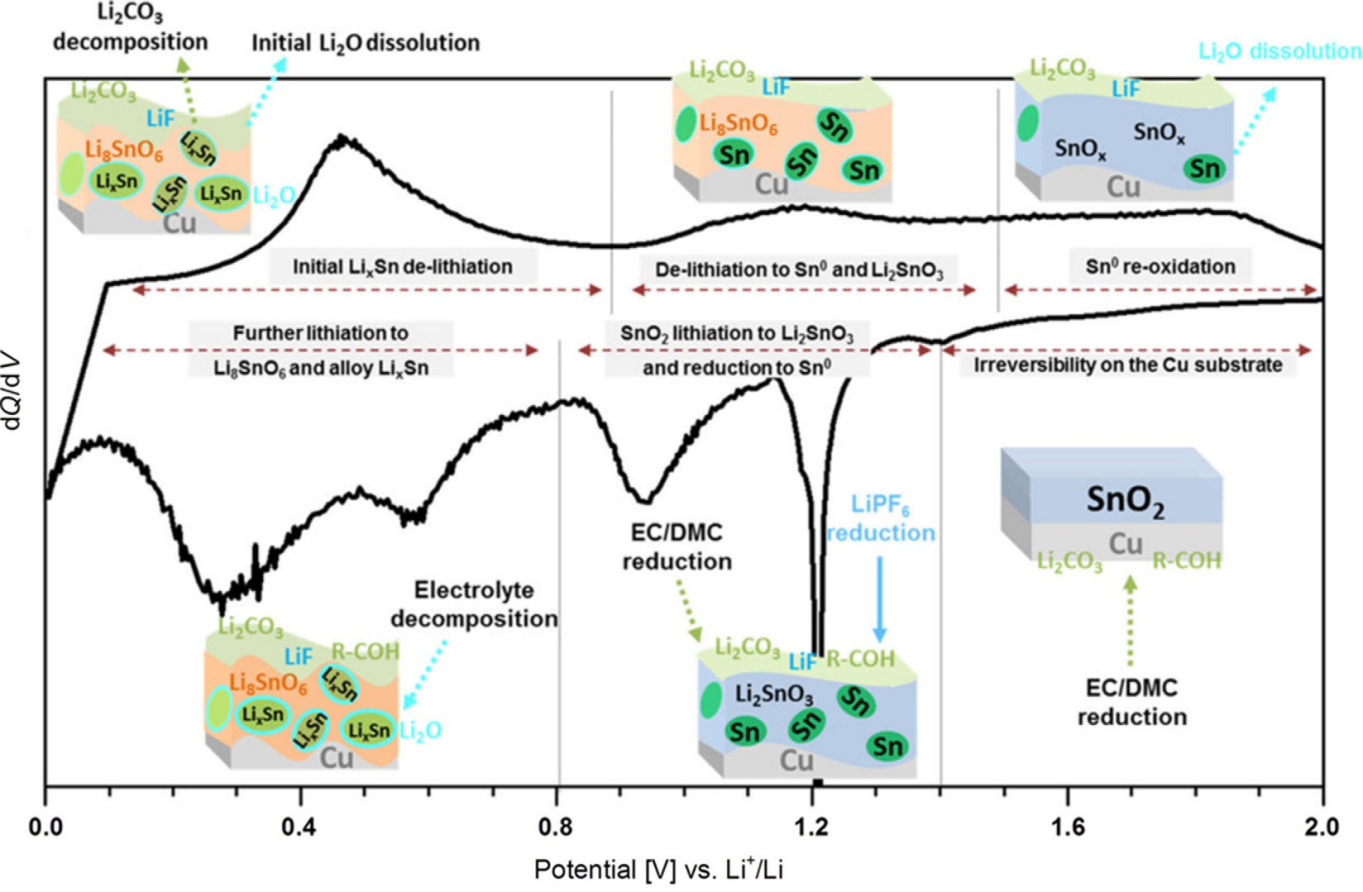
Cyclic voltammogram of a flat SnO_2_ model electrode with a schematic representation of the electrode composition, intermediate phases during lithiation, and redox features associated with interfacial reactions with the organic and inorganic part of the electrolyte. EC=ethylene carbonate, DMC=dimethyl carbonate. Reproduced with permission from Ref. 6. Copyright 2018, American Chemical Society.

However, more recent theoretical calculations^7^ and in situ scanning transmission electron microscopy on nanowires7b suggested the occurrence of Li^+^ insertion into the SnO_2_ lattice preceding the abovementioned steps (Figure 1).

Based on the latest findings, the total process of the lithium reaction with SnO_2_ can be presented as Equations (1), (2), (3).

(1) $$\mathrm{insertion}\;\;(\mathrm{intermediate}\;\;\mathrm{phase}):\mathrm{SnO}{}_{2}+x\;\mathrm{Li}{}^{+}+x\;e{}^{-}\vec{\leftarrow }\mathrm{Li}{}_{x}\mathrm{SnO}{}_{2}$$

(2) $$\mathrm{conversion}:\mathrm{SnO}{}_{2}+4\;\mathrm{Li}{}^{+}+4\;e{}^{-}\to \mathrm{Sn}+2\;\mathrm{Li}{}_{2}O>1.2\;V\;\;\mathrm{vs}.\;\;\mathrm{Li}/\mathrm{Li}{}^{+}\;\;\mathrm{with}\;\;\approx 711\;\mathrm{mAh}\;g{}^{-1[6]}$$

(3) $$\mathrm{alloying}/\mathrm{dealloying}:\mathrm{Sn}+x\;\mathrm{Li}{}^{+}+x\;e{}^{-}\to \mathrm{Li}{}_{x}\mathrm{Sn}\;\;\left(0\leq x\leq 4.4\right)<0.5\;V\;\;\mathrm{vs}.\;\;\mathrm{Li}/\mathrm{Li}{}^{+}\approx 783\;\mathrm{mAh}\;g{}^{-1[6]}$$

The Li_*x*_SnO_2_ intercalation compound [according to Eq. (1)] is an intermediate phase formed by long‐range Li^+^ diffusion into the SnO_2_ phase mediated by the nucleation of dislocations.7b Ab initio calculations for the first lithiation cycle predicted Li_2_SnO_3_ and Li_8_SnO_6_ as compositions of intermediate phases.7a Recently, Ferraresi et al. found strong experimental evidence for the existence of these phases by combining electrochemistry, postmortem X‐ray photoelectron spectroscopy (XPS), and SEM imaging together with DFT calculations.^6^ The few available reports in the literature indicate that the composition and spatial distribution of intermediate Li−Sn−O phases and the reversibility of subsequent reactions steps are strongly affected by the composition and morphology of parent SnO_2_ electrodes. The crystallinity and composition (exact stoichiometry, defects, surface termination, impurities), as important parameters of SnO_2_ materials, all influenced by the choice of precursors and the fabrication method, are known to affect their electrochemical performance and stability. Studies on a flat amorphous SnO_2_ film as a model electrode demonstrate that the reversibility of the reaction steps strongly depends on the reactions during the first lithiation cycle, as proposed by calculations on the Li_*x*_Sn phase diagram.^6^, ^7^ The typical cyclic voltammogram (Figure 1) furthermore shows redox features of side reactions at the interface that are associated with solid–electrolyte interface (SEI) formation and electrolyte reduction, which contribute to irreversible capacity loss (ICL) of SnO_2_‐based anodes in the first cycles.^6^

In a subsequent conversion reaction [Eq. (2)], the intermediate Li_*x*_SnO_2_ compounds are reduced to metallic Sn, which crystallizes in a Li_2_O matrix.^7^ The conversion reaction of SnO_2_ to metallic tin is reported to be irreversible for bulk SnO_2_, but it can be (partially) reversible for nanosized SnO_2_; this greatly depends on the particle size and morphology.4b, 5a, 8

**Figure 2:**
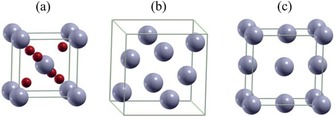
Schematic representation of unit cells of a) SnO_2_ (rutile *P*4_2_ *mnm*), b) α‐Sn (cubic *Fd*3*m*), and c) β‐Sn (*I*4_1_/*amd*). Gray and red spheres represent Sn and O atoms, respectively. Reproduced (adapted) with permission from Ref. 7a. Copyright 2015, Royal Society of Chemistry.

Upon further Li‐ion uptake, the surrounding matrix with metallic Sn particles is lithiated to form Li_*x*_Sn alloys [Eq. (3)]. It has been shown that, starting from the β‐Sn phase, a mixture of cubic α‐ and the tetragonal β‐Sn (Figure 2 b,c) is formed; the α‐phase is stabilized for small nanostructures.7a, 9 The alloying/dealloying process between Sn and Li^+^ is considered to be reversible.8c, 8d

**Figure 3:**
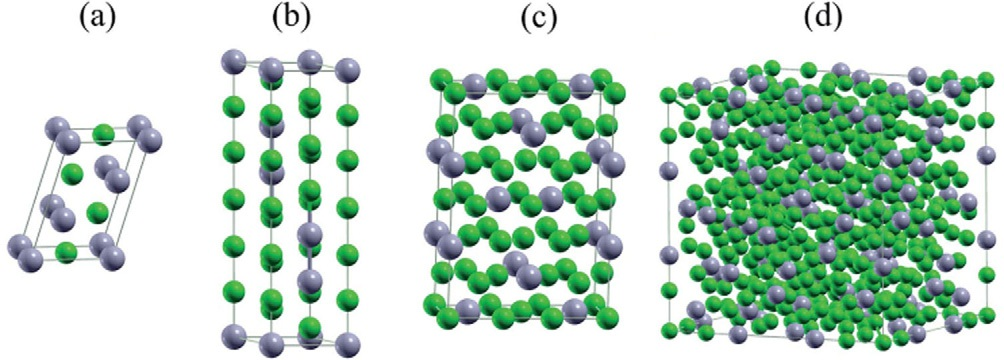
Schematic representation of theoretically predicted intermediate Li_*x*_Sn alloys: a) LiSn, b) Li_13_Sn_5_, c) Li_7_Sn_2_, and d) Li_17_Sn_4_. Green and gray spheres represent Li and Sn atoms, respectively. Reproduced (adapted) with permission from Ref. 7a. Copyright 2015, Royal Society of Chemistry.

According to experimentally determined and ab initio calculated Li_*x*_Sn phase diagrams, the following Li−Sn alloys are proposed to form during the lithiation/delithiation cycles with increasing Li content: LiSn, Li_13_Sn_5_, Li_7_Sn_2_, up to Li_17_Sn_4_ (Figure 3 a–d).7a

The specific capacity of the SnO_2_ anodes is greatly dependent on the reversibility of different reaction steps. The theoretical capacity of the complete reaction, including both conversion and alloying is as high as 1494 mAh g^−1^, but it reduces to 783 mAh g^−1^ if only the alloying/dealloying reaction is reversible. It should be noted, however, that, even if only partial reversibility of the alloying/dealloying step is possible, the specific capacity still significantly exceeds that of graphite (372 mAh g^−1^).^10^

Apart from the quasi‐irreversibility of the conversion reaction and subsequent severe capacity loss during the first cycles, SnO_2_‐based anodes suffer from large volume changes of up to 250 % during the alloying and dealloying process.5c This causes internal stress that leads to pulverization of the electrode. Moreover, in situ XRD and TEM measurements also reveal that the formed tin particles can agglomerate into tin clusters that are less electrochemically active. Last, but not least, volume changes impede the formation of a stable SEI, which prevents further electrolyte decomposition. These factors are responsible for fast capacity fading and decreased cycling performance upon repeated cycling,5a, 8a, 8c, 8d, 9, 10 and are the main reasons that SnO_2_‐based anodes have not yet been commercialized.

The shortcomings of SnO_2_‐based anodes are mainly addressed by using two main strategies. One is to tailor bulk SnO_2_ down to the nanosize and/or to nanostructure the SnO_2_ compounds towards nanoparticles,^9^, ^11^ 1D nanorods,^12^ nanowires,^13^ nanotubes,^14^ 2D nanosheets,^15^ and 3D porous^16^ or hollow8d, 17 structures. Nanosized materials are known to accommodate large volume changes and to shorten diffusion paths for electrons and lithium ions. Porous or hollow structured (nanosized) SnO_2_ can provide additional free space to reduce the problems of pulverization and large volume changes.1b

Another effective approach is the fabrication of composites of SnO_2_ and carbonaceous materials. The carbonaceous supports increase the overall conductivity of the composites and can also buffer large volume changes of SnO_2_ during alloying and dealloying. There are many reports on carbon coating of SnO_2_,^18^ as well as composites consisting of SnO_2_ and carbonaceous materials, including carbon nanotubes (CNTs),^19^ fibers,^20^ aerogels,^21^ hollow spheres,^22^ and graphene.^23^

Herein, we introduce recent developments regarding different tin oxide based anode materials systems, with a focus on the properties of the materials that affect their application in future energy‐storage devices. Based on the analysis of key electrochemical properties, the phases identified during electrochemical transformations and the consequences arising for the reversibility of their transformations, the general goal of this Minireview is to indicate solutions to maximize the initial storage capacity and to overcome ICL, which is mainly associated with the conversion reaction. The most promising strategies to improve the performance of SnO_2_‐based anodes, such as nanostructuring, doping, and composite formation, to obtain high‐rate and high‐capacity anodes for future LIBs, and potentially also for sodium‐ (NIBs) and potassium‐ion batteries (KIBs), are discussed in separate sections.

## 2 Nanostructured Phase‐Pure SnO_2_ LIB Anodes

Large volume changes, together with repeated cycling of bulk SnO_2_, leads to pulverization of the anode and to decreased electrical contact, which causes a drastic loss in capacity within a few cycles. Other serious drawbacks of pure SnO_2_ are its low electronic and ionic conductivity. A very low room‐temperature conductivity of SnO_2_ of 1.82×10^−8^ S cm^−1[24]^ drastically limits its storage and rate capability as an anode material. The measured apparent lithium‐ion diffusion coefficient is also low; the reported values range from 10^−16^–10^−14^ cm^2^ s^−1^ for a sputtered metallic Sn film (3 μm thick) to 10^−15^–10^−13^ cm^2^ s^−1^ for amorphous SnO_2_ tin oxide films (≈1.5 μm).^25^

Similar to other electrode materials with comparable properties (Si can be mentioned as an important example), nanostructuring is considered to be a promising strategy to mitigate the intrinsic drawbacks of the materials. Nanocrystalline SnO_2_, with various nanomorphologies, can accommodate volume expansion through built‐in porosity and reduce the agglomeration of Sn clusters by a homogeneous dispersion within an Li_2_O matrix. It can furthermore decrease the required Li^+^ diffusion pathway by a significantly increased electrode–electrolyte interface, and thereby enable a higher flux of ions, resulting in high rate‐capable anodes.1a, 26 In addition, nanostructured SnO_2_ may display altered properties, depending on the synthetic conditions, such as a significantly increased electrical conductivity of 0.1–0.9 S cm^−1^ measured on single nanorods^27^ or the preservation of nanocrystallinity indicated by the presence of an α‐Sn phase upon repeated cycling.7a, 9 The presence of a nanocrystalline α‐Sn phase is thereby correlated to the reversibility of the alloying reaction; however, it is not clear whether it is actually the phase that influences reversibility. The α‐phase, which is more stable on a nanoscale, might indicate the intactness of the initial nanomorphology and, particularly, the fine distribution of Sn within the Li_2_O matrix, which is important for reversibility.

A comprehensive review, with a focus on synthetic routes and electrochemical performance of phase‐pure SnO_2_‐based anodes, was published by Chen and Lou in 2013.1a Hence, we aim to provide an update on recent developments of SnO_2_‐based nanostructures applicable as anodes in LIBs and to link the properties of materials and initial SnO_2_ morphologies defined by the synthetic parameters with electrochemical performance and stability of the resulting electrodes.

### 2.1 Nanoparticles

**Figure 4:**
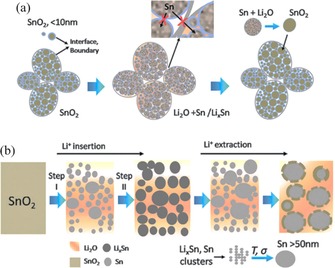
Schematic representation of SnO_2_ anode microstructures formed in the course of de‐/lithiation cycles and resulting structural changes. a) Structural evolution of a hierarchically nanostructured SnO_2_ electrode, with a large number of interfaces and grain boundaries that inhibit Sn/Li_*x*_Sn coarsening and allow for a reversible conversion reaction. b) Structural evolution of the initial conversion and first lithiation cycle of bulk (>50 nm) SnO_2_ electrode that permits a quantitative reversible conversion reaction. Reproduced (adapted) with permission from Ref. 4b. Copyright 2016, Royal Society of Chemistry.

Diffraction studies on SnO_2_ anodes revealed an ICL due to the formation of the amorphous Li_2_O matrix and afterwards the loss in reversible capacity upon cycling. The reversibility of the reaction upon cycling was correlated to the initial SnO_2_ crystallite size.^28^ Generally, it can be said that only if the active Sn material resulting from the conversion of nanosized SnO_2_ crystals is well dispersed in the amorphous Li_2_O matrix is a reversible alloying reaction without drastic capacity fading possible (Figure 4 a). Larger Sn particles that are not homogeneously dispersed in the amorphous Li_2_O matrix aggregate to form even larger clusters upon cycling, which leads to mechanical and electronic disintegration of the electrode (Figure 4 b).4b, 28

In 2004, Ahn et al. reported SnO_2_ particles about 11 nm in size, which were prepared through a colloidal method, to be an optimum size for lithium storage and reversibility with respect to the alloying reaction.8a In contrast, even smaller SnO_2_ nanoparticles (e.g., 2 nm) have shown a high ICL. As a possible reason, increased SEI formation on very small nanoparticles, due to their larger electrochemical surface area, as well as a decreased formation of the surrounding Li_2_O phase, were proposed; this may lead to increased aggregation, and thus, capacity fading.8a Conclusions about the optimum particle sizes are, however, not corroborated by other reports and seem to be strongly influenced by the synthetic route. Thus, Kim et al. reported that hydrothermally synthesized particles of about 3 nm in size showed an optimum initial (≈740 mAh g^−1^ at 60 mA g^−1^ for the first cycle discharge current) and reversible capacity and cycling stability (negligible fading over 60 cycles at 300 mA g^−1^ discharge current).^9^ It can be suggested that the optimum size of SnO_2_ nanocrystals, with respect to reversible capacity and capacity retention, is strongly dependent on the exact nature and amount of amorphous Li_2_O matrix surrounding Sn formed during the initial conversion reaction, which is, in turn, affected by the SnO_2_ nanoparticle synthetic route and initial cycle lithiation parameters (see also the discussion about the reaction mechanism in the Introduction).

A recent study by Hu et al. suggested that the capacity decay of SnO_2_‐based electrodes with larger nanoparticles was not directly induced by mechanical disintegration of the electrode due to large volume changes, but associated with a gradual degradation of the reversible conversion reaction at potentials below 1.0 V versus Li/Li^+^.^10^ Thermal and stress‐driven Sn coarsening that could be correlated to the average crystallite size has been identified as a main factor responsible for the reversibility of the conversion reaction, and thus, the reversible capacity of SnO_2_‐based electrodes. Furthermore, a quantitative relation between Sn‐grain coarsening and the initial SnO_2_ crystallite size was found, with a critical size of 11 nm for a fully reversible conversion reaction. Smaller crystallites with high‐density Sn/Li_2_O interfaces are reported to possess fast enough interdiffusion kinetics that enable a fully reversible conversion reaction.

Through their synthetic approach based on magnetron‐sputtered pure SnO_2_ thin films, Hu et al. obtained an initial capacity of 1066 mAh g^−1^, with a reversible capacity of about 915 mAh g^−1^ at a rate of 0.2 A g^−1^ after 20 cycles, which remained stable for over 100 cycles followed by a slow decay.^10^

A further recent example of SnO_2_ nanoparticles includes the fast and scalable microwave‐assisted hydrothermal synthesis of fine particles of about 14 nm in size. An initial discharge capacity of about 1197 mAh g^−1^, with a reversible capacity of 520 mAh g^−1^ (2nd cycle), and a capacity retention of about 53 % (273 mAh g^−1^) after 50 cycles at 100 mA g^−1^ were reported for this material by Yin et al.11a

Jiang et al. demonstrated a large‐scale hydrothermal synthesis of SnO_2_ nanoparticles about 6 nm in size.11b Fabricated anodes that were cycled between 0.01 and 3.0 V versus Li/Li^+^ showed an initial discharge capacity of 2223 mAh g^−1^ at a rate of 0.1 A g^−1^ with a fast capacity fading to about 800 mAh g^−1^ within the first 20 cycles and a slow decay to 760 mAh g^−1^ after 40 cycles.11b The reported capacity outperforms the values published for other morphologies, such as nanosheets, ‐tubes, ‐rods, or ‐spheres, and is in the range of tin oxide based carbon and transition‐metal oxide composites.

**Figure 5:**
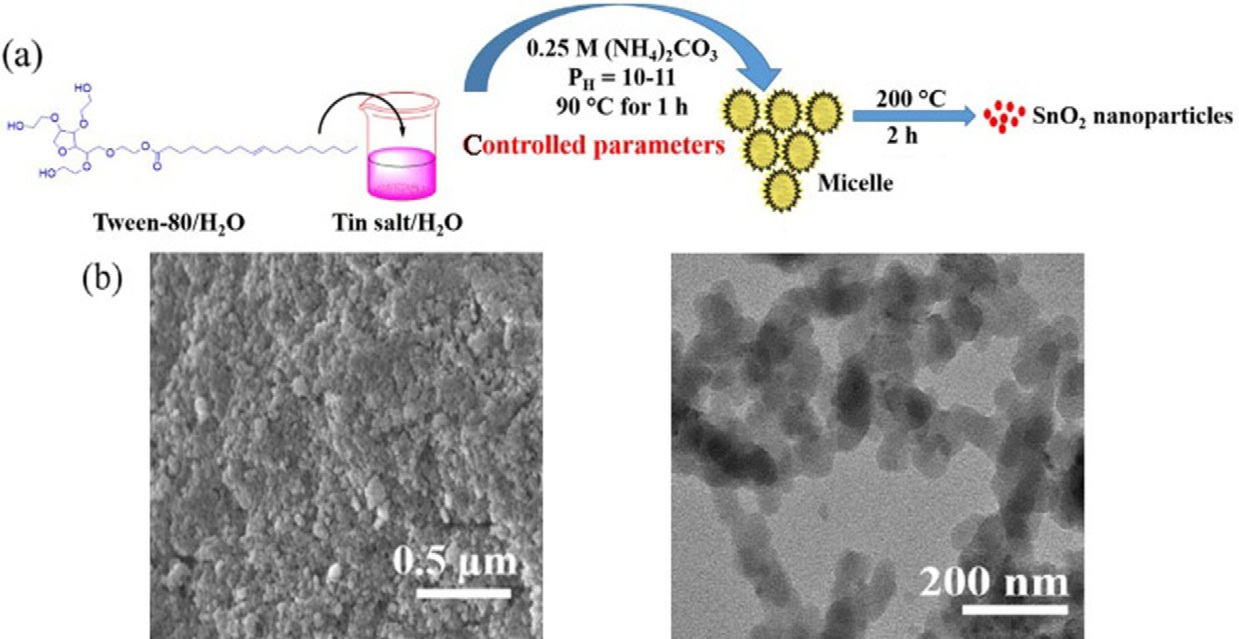
Schematic representation of the synthesis of porous SnO_2_ electrode materials built from nanoparticles. a) Tween‐80 surfactant‐mediated synthesis of SnO_2_ nanoparticles. b) SEM and TEM images of porous SnO_2_ consisting of interconnected nanoparticles. Reproduced (adapted) with permission from Ref. 29. Copyright 2017, Elsevier.

To enhance the rate capability and lithium‐storage capacity of SnO_2_‐based anodes, Hameed et al. used a hydrothermal synthetic method with the micelle‐forming surfactant Tween‐80 to obtain mesoporous powders of connected SnO_2_ nanoparticles (Figure 5) or ‐rods.^29^ The resulting electrodes showed an initial discharge capacity of 1877.8 mAh g^−1^, with fast capacity fading within the first 20 cycles to stabilize with prolonged cycling at 641.1 mAh g^−1^ at a high discharge rate of 200 mA g^−1^ (doubled in comparison to the majority of examples reported in the literature). The rate capability of the porous nanoparticle electrodes is thereby outstanding, with values of 629, 490, and 340 mAh g^−1^ at current densities of 300, 500, and 1000 mA g^−1^, respectively; this is attributed to their open and accessible morphology.^29^

Apart from 0D structures, in the form of nanoparticles, considerable effort was made, in recent years, to fabricate anodes with diverse 1D to 3D morphologies. The goal is to form an optimized electrode–electrolyte interface that enables fast lithium diffusion kinetics from the electrolyte, but also a maximized utilization of active material by offering short diffusion pathways in nanostructures. The second aim is to fabricate “breathable” structures that can accommodate volume changes induced by the alloying/dealloying reaction during cycling, and therefore, prevent mechanical and electrical disintegration of the active material.

### 2.2 Nanorods

The synthesis of high aspect ratio SnO_2_ structures was initially demonstrated by Liu et al. in 2001 for an inverse microemulsion system (IμE).^30^ The reaction conditions, including the choice of precursors and a high calcination temperature (≈800 °C), are thereby similar to those used in the molten salt synthetic method widely applied for the formation of nanostructured ceramic powders.

Since then, several groups have adapted the concept of IμE‐based synthesis; first with a high or moderate temperature and/or salt‐assisted calcination and later also by using a solvo‐/hydrothermal approach at temperatures as low as 150 °C.1a, 12, 31

In 2010, Xi and Yi synthesized nanorods with diameters down to 1–1.5 nm that exhibited a strong quantum confinement effect, increasing *E*_g_ by about 0.9 eV relative to that of bulk SnO_2_.^32^ A main focus of the work, however, was to investigate the nanorod growth mechanism through time‐dependent diffraction and high‐resolution (HR) TEM measurements.

According to Equation (4), the formation of sphere‐like SnO_2_ nanoparticles is driven by a mild hydrolysis reaction (aqueous urea solution at 90 °C):

(4) $$\mathrm{Sn}{}^{4+}+4\;\mathrm{OH}{}^{-}\to \mathrm{Sn}\left(\mathrm{OH}\right){}_{4}\to \mathrm{SnO}{}_{2}+2\;H{}_{2}O$$

Larger cubelike SnO_2_ nanoparticles with defined crystal facets evolve from a classical crystallization process known as Ostwald ripening. The 1D nanorod morphology is then obtained without templating agents or long‐chain organic solvents through an energetically driven assembly of particles on their (001) facets to reduce the surface energy, ultimately leading to a growth along the [001] orientation. These 1D aggregates of SnO_2_ nanoparticles recrystallize to finally form single‐crystalline SnO_2_ nanorods.^32^

**Figure 6:**
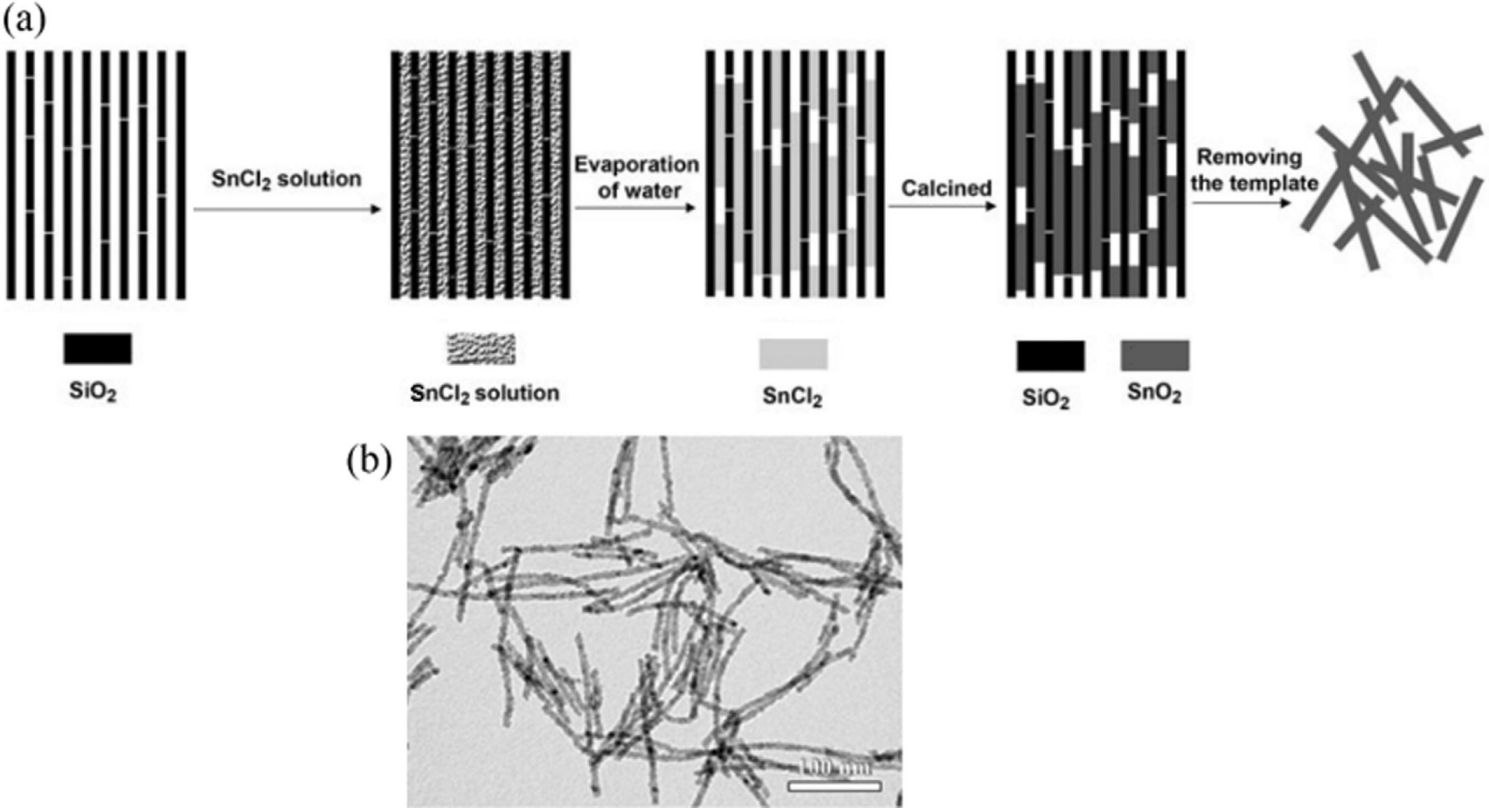
Schematic representation of the proposed mechanism for the formation of rodlike SnO_2_ by using a mesoporous SBA**‐**15 silica template (a), along with a transmission electron micrograph of the product (b). Reproduced (adapted) with permission from Ref. 33. Copyright 2014, Materials Research Society.

Examples of the nanorod morphology employed in SnO_2_‐based anodes in recent years include the synthesis of SBA‐15‐templated active material by Jiao et al. in 2014.^33^ In this work, a solution of SnCl_2_ is used for the infiltration of a mesoporous silica (SBA‐15) hard template, which is removed after drying and calcination of the SnO_2_ nanorods inside its aligned pores (Figure 6).

The resulting anode material showed an initial discharge capacity of 1119 mAh g^−1^ and a reversible capacity of about 700 mAh g^−1^ (2nd cycle) that declined to about 300 mAh g^−1^ within 50 cycles at a rate of 100 mAh g^−1^, which corresponded to a capacity retention of about 43 %.^33^

In 2015, Han et al. synthesized larger, highly aligned SnO_2_ nanorods in the size range of about 50×100–150 nm on a self‐produced Na_2_Sn(OH)_6_ substrate through a one‐step, template‐free hydrothermal synthetic method.^34^ Single‐crystalline rods grew along the [001] orientation on the substrate and exposed (110) facets. An initial discharge capacity of 1930 mAh g^−1^ was determined for this material, with a high reversible capacity of around 1000 mAh g^−1^ that was retained at about 60 % at a rate of 100 mA g^−1^ after 20 cycles (≈600 mAh g^−1^).

**Figure 7:**
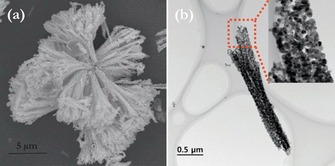
SEM (a) and TEM (b) images of hydrothermally prepared SnO_2_ nanorod bundle(s). Reproduced (adapted) with permission from Ref. 35. Copyright 2017, Elsevier B.V.

In 2017, Sennu et al. used a modified precipitation route, with a related mild hydrothermal treatment and calcination, to obtain bundles of SnO_2_ nanorods with dimensions of 2–3.5 and 0.2–0.3 μm in length and diameter, respectively.^35^ The material morphology resembling marine algae is polycrystalline in nature and built up from individual SnO_2_ particles of around 10–20 nm (Figure 7).

In corresponding half‐cell measurements, a high initial discharge capacity of 2697 mAh g^−1^ was measured. A reversible capacity of about 695 mAh g^−1^ fades within 50 cycles to reach about 650 mAh g^−1^, which represents a remarkably high capacity retention of about 94 % (scan rate of 100 mA g^−1^ and 24 wt % conductive additive).

### 2.3 Nanowires and ‐tubes

SnO_2_ conversion and alloying anodes with 1D nanowire morphology were fabricated by various synthesis approaches in recent years.

Wu et al. synthesized nanowires of about 200 nm in diameter and several micrometers in length through a carbon‐assisted thermal evaporation technique under ambient conditions in a single zone tube furnace.^36^ A promising initial reversible capacity of about 1350 mAh g^−1^, with a capacity retention of about 46 % (≈620 mAh g^−1^) after 50 cycles, was achieved at 100 mA g^−1^.

Lee and Kim synthesized SnO_2_ nanowire arrays by means of chemical vapor deposition (CVD) with distinct patterns by using a photolithographic process.13b The best performing samples of this type showed an initial discharge capacity of about 1600 mAh g^−1^ and a reversible capacity of about 700 mAh g^−1^ that faded to about 500 mAh g^−1^ within 50 cycles (≈71 % capacity retention), and down to 400 mAh g^−1^ within 100 cycles.

In 2017, Lee et al.13a were able to synthesize hierarchically branched SnO_2_ nanowires through a two‐step CVD method, which showed a slightly increased performance compared with that of the work of Lee and Kim.13b The material also showed initial discharge and reversible capacities of about 1600 and 800 mAh g^−1^, respectively, with 69 % capacity retention (≈550 mAh g^−1^) after 50 cycles, and about 400 mAh g^−1^ after 100 cycles at a rate of 0.1 C (1 C=400 mA g^−1^).13a

**Figure 8:**
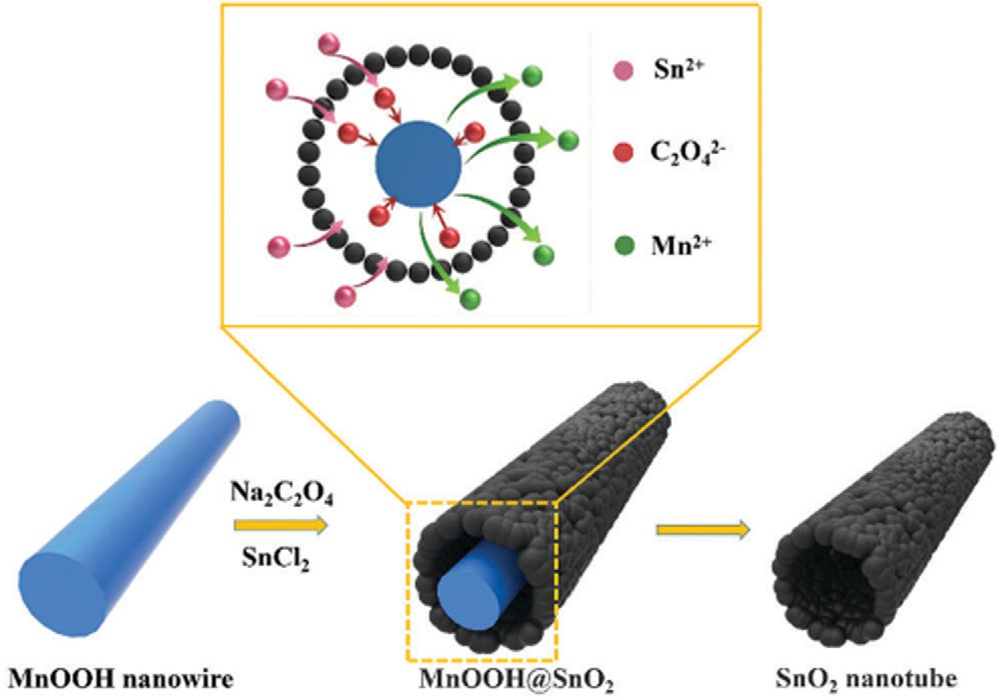
Schematic illustration of the formation of SnO_2_ nanotubes through an oxalate‐assisted redox etching and precipitating′ route. Reproduced with permission from Ref. 37. Copyright 2017, The Royal Society of Chemistry.

Related nanotube SnO_2_ morphologies were recently investigated by Han et al. in an oxalate‐assisted “redox etching and precipitating”′ route involving MnOOH nanowires and Sn^2+^ ions. SnO_2_ nanotubes with a diameter of 200–250 nm and several micrometers in length were synthesized (Figure 8).^37^

Electrode measurements showed an initial discharge capacity of about 2000 mAh g^−1^ with a high reversible capacity of 1400 mAh g^−1^ that faded to 700 mAh g^−1^ within 50 cycles (50 % capacity retention). Extended cycling showed a rather high stability of the electrode material, with a discharge capacity of 500 mAh g^−1^ after 100 cycles at an elevated rate of 500 mA g^−1^.^37^

### 2.4 Nanosheets

The 2D SnO_2_ nanosheet morphology and its application as a LIB anode material was thoroughly discussed in a review by Chen and Lou in 2012.^38^

The electrochemical performance of nanosheet‐based anodes was found to be greatly influenced by the morphology, crystallinity, and phase purity of SnO_2_, with a strong effect of the precursors used on the resulting product. Thus, anisotropic growth of SnO_2_ with the formation of nanosheets was successfully achieved through hydrothermal synthesis with SnCl_2_ as the precursor.^38^ However, the presence of fluoride ions, either by using SnF_2_ as the tin oxide precursor or by using an additional fluoride source, such as NH_4_F, with the actual tin oxide precursor (e.g., SnCl_2_) was shown to lead to the formation of an SnO/SnO_2_ mixture (for SnF_2_ as the precursor) or phase‐pure SnO_2_ nanosheets (for NH_4_F as an additive), respectively, under hydrothermal conditions.^39^

**Figure 9:**
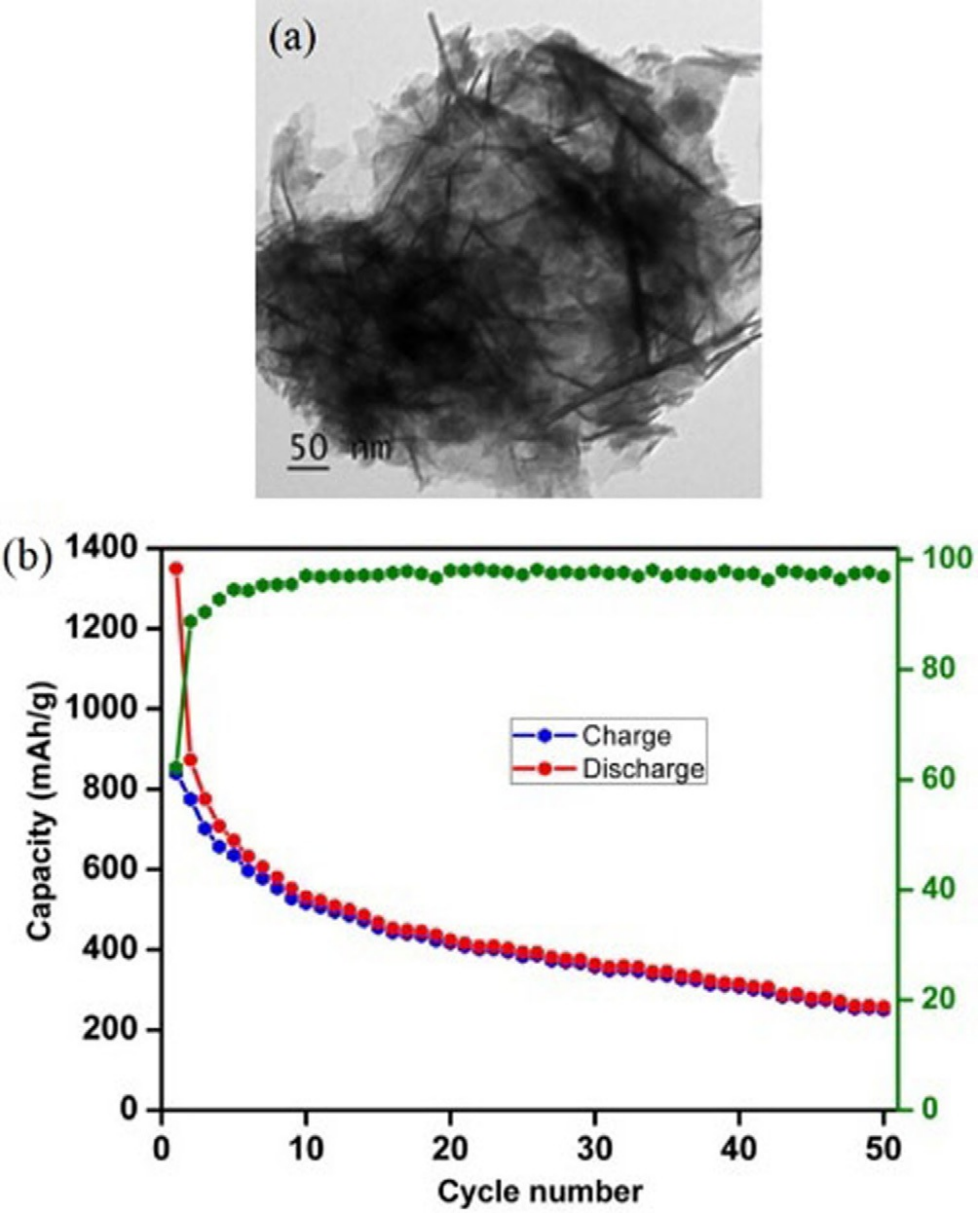
a) TEM image of agglomerated SnO_2_ nanosheets and b) galvanostatic charge/discharge curves of SnO_2_ nanosheet‐based LIB anodes at a current density of 100 mA g^−1^. Reproduced (adapted) with permission from Ref. 15a. Copyright 2017, Elsevier and Techna Group.

A recent example for the fabrication of SnO_2_ nanosheets is given by the work of Narsimulu et al., who described the surfactant‐ and template‐free hydrothermal and microwave‐assisted synthesis of agglomerated SnO_2_ nanosheets (Figure 9).15a The respective electrodes showed a moderate initial discharge capacity of 1350 mAh g^−1^, with a reversible capacity of 873 mAh g^−1^ that faded to 258 mAh g^−1^ within 50 cycles at a rate of 100 mA g^−1^.15a

### 2.5 3D hollow nanostructures

Beyond the 0D, 1D, and 2D SnO_2_ materials introduced above, porous 3D morphologies were fabricated in recent years. Among them, hollow and porous nano‐ and microspheres,16a, 17 as well as 3D ordered macroporous structures,^40^ were synthesized and proposed to possess structural flexibility to counteract fast pulverization of the anode by volume changes induced upon cycling.

**Figure 10:**
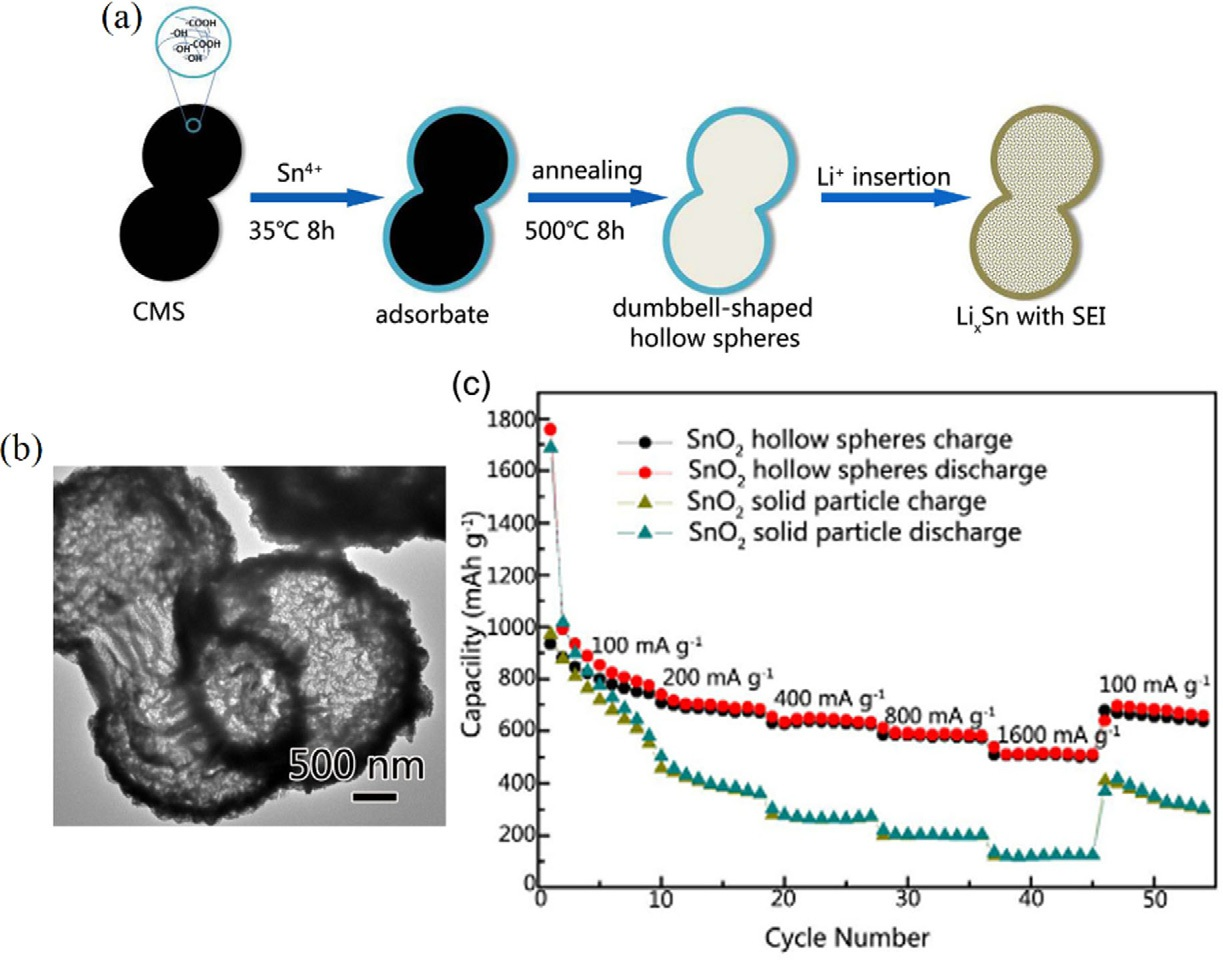
a) Schematic representation of the synthesis of dumbbell**‐**shaped hollow spheres. b) TEM image and c) galvanostatic charge/discharge curves at varying rates (100**–**1600 mA g^**−**1^). Reproduced (adapted) with permission from Ref. 16a. Copyright 2018, Elsevier.

A promising synthetic route was presented by Li et al., who used negatively charged carbonaceous microspheres (CMSs) prepared through a hydrothermal method that electrostatically bound Sn^4+^ ions on their surface.16a After calcination in air with simultaneous template removal, hollow dumbbell‐shaped microspheres of several micrometers were obtained (Figure 10).

Electrochemical measurements reveal a very high and reversible lithium‐ion storage capability of the material of about 1000 mAh g^−1^ in the second discharge cycle (≈1750 mAh g^−1^ in the first discharge cycle) that is maintained after 100 cycles, with about 600 mAh g^−1^ at a rate of 500 mA g^−1^ and still above 500 mAh g^−1^ with an applied rate of 1 A g^−1^. The capability of the hollow spherical structures to effectively buffer volume changes and to allow high rate applications is reflected by galvanostatic charge/discharge measurements, with rates of up to 1600 mA g^−1^ and a resulting capacity of over 500 mAh g^−1^, which returns to 695 mAh g^−1^ if the rate is decreased to the initial value of 100 mA g^−1^.16a

Another way to obtain large hollow SnO_2_ structures with rodlike shapes was developed by Wang et al.^41^ In their synthetic approach, a genetically modified microbial *Escherichia coli* template binds a Sn^2+^ precursor on its surface through electrostatic interactions. Subsequent calcination results in the formation of about 400×600 nm rodlike hierarchical SnO_2_ structures composed of smaller nanosheets and particles. However, the electrochemical performance of the prepared anodes is moderate, with an initial discharge capacity of about 975 mAh g^−1^ and a capacity retention fading to 194 mAh g^−1^ (≈20 % of the initial value) after 50 cycles at a rate of 200 mA g^−1^.

## 3 Doped SnO_2_ LIB Anodes

Element doping is a known approach to optimize the electrochemical performance of SnO_2_‐based electrodes. Doping can lead to a greatly increased electronic conductivity, which is beneficial for the electrode performance.

Pure SnO_2_ is a wide band gap semiconductor, with an optical band gap of 3.6 eV at room temperature. It exhibits an intrinsic n‐type conductivity due to the presence of shallow donor levels located at 0.46 eV below the conduction band, which is attributed to ionized defects (e.g., unintentional hydrogen atom doping), according to computational studies by Singh et al. and more recently by Villamagua et al.^42^ Fluorine doping is reported to increase the conductivity up to about 5×10^3^ S cm^−1^ by substituting O^2−^ in the crystal structure, and thereby creating shallow donors that enhance the n‐type conductivity significantly.^43^

Due to better synthetic control than that with fluorine doping, p‐type doping with Group III atoms (In, Ga, and Al)42b or n‐type doping with Group V atoms (Sb doping),^44^ which creates shallow levels, was thoroughly explored in recent years. In addition to increasing conductivity, transition‐metal doping is reported to decrease large volume changes upon lithiation/delithiation.^45^ In recent years, a variety of transition‐metal dopants for SnO_2_ were proposed in the literature; these can be divided into two groups: redox‐inactive and ‐active elements that can undergo conversion/alloying reactions with lithium ions in the potential range applicable for SnO_2_‐based anodes.^46^ Niobium,^47^ titanium,^48^ zirconium,^46^ palladium,^49^ and tungsten^50^ can be assigned to the first group. Doping with these transition‐metal ions does not result in an observable gain in capacity because the lithiation/delithiation curves of SnO_2_ anode materials remain unchanged, without additional redox features from the doping elements in the respective potential window. However, doped tin oxides show a significantly increased cyclability and rate capability.^46^ The beneficial effect on the cycling performance provided by both redox‐active and ‐inactive transition‐metal doping in conversion‐type anodes (ZnO, SnO_2_) was initially attributed to the decreased crystal size observed upon doping; thus limiting the aggregation of primary nanoparticles and enabling a reversible lithium alloy formation.^51^ Recent investigations suggest that the improved performance of doped tin oxides results from an increase in the conductivity of the active material caused by an additional charge percolation pathway provided by the transition‐metal (dopant) ion network in the SnO_2_ structure, as well as through an increase in the intrinsic conductivity through newly generated surface oxygen vacancies.^49^, ^50^, ^51^ The degree of conversion reaction versus side reactions, such as particle aggregation, is thereby correlated with the reaction kinetics, which depend strongly on the electron‐transfer properties and local current density.^49^, ^50^ Apart from increased conductivity, a catalytic effect of transition‐metal ions on decomposition of the Li_2_O phase is discussed; this further promotes a reversible conversion reaction.^49^ In the context of widely applied SnO_2_/graphene composites, transition‐metal doping (W‐doped SnO_2_) has also been shown to reduce the charge‐transfer resistance between active material particles and graphene through an increased interaction at the interface.^50^

Redox‐active dopants include manganese,^46^, ^52^ iron,^46^, ^52^, ^53^ antimony,44b, 54 cobalt,4a, 45a, 45c, 45d, 46, 52, 55 nickel,^46^ copper,^46^ zinc,45d, 46, 56 and molybdenum.45b In addition to the effect of redox‐inactive dopants discussed above, their corresponding metal oxides can, in principle, undergo a conversion reaction with lithium over the applied potential range of the anode, resulting in a theoretical gain in capacity (see also Section 4.4).^46^ However, the increased capacity does not necessarily translate into an increased energy density of a full‐cell assembly because dopants (e.g., Cu) can cause a voltage hysteresis; thus lowering the total storable energy.^46^

Moreover, other dopants or multidoping strategies have been reported, for example, Mg,45d Al,^57^ In,^58^ F,45c, 59 N,^60^ P,^61^ S/F,^62^ Co/F,45c and Co/N.45a

Among others, cobalt is an interesting redox‐active dopant because Co‐doped SnO_2_ shows a volume buffering effect that is attributed to a reduced and maintained small SnO_2_ primary particle size upon cycling. Furthermore, Co‐doped SnO_2_ demonstrates a measurable gain in capacity versus undoped SnO_2_, with a decreased voltage hysteresis and increased coulombic efficiency.45a, 45d Nithyadharseni et al. compared Co‐, Mg‐, and Zn‐doped SnO_2_ nanoparticles.45d The compounds were prepared through sol–gel synthesis with ethylene glycol, dimethyl ether, and citric acid. They found that cobalt doping led to a superior electrochemical performance. The Co‐doped electrodes deliver a specific capacity of 573 mAh g^−1^, compared with 330 mAh g^−1^ for the undoped sample, after 50 cycles at 60 mA g^−1^. They attributed this to structural stability and Co−Sn intermetallic interactions. Lübke et al. reported similar results and confirmed that, in their case, Co doping was also superior to that of Nb‐, Ti‐, Zr‐, Fe‐, Cu‐, Zn‐, Mn‐, and Ni‐doped materials.^46^

**Figure 11:**
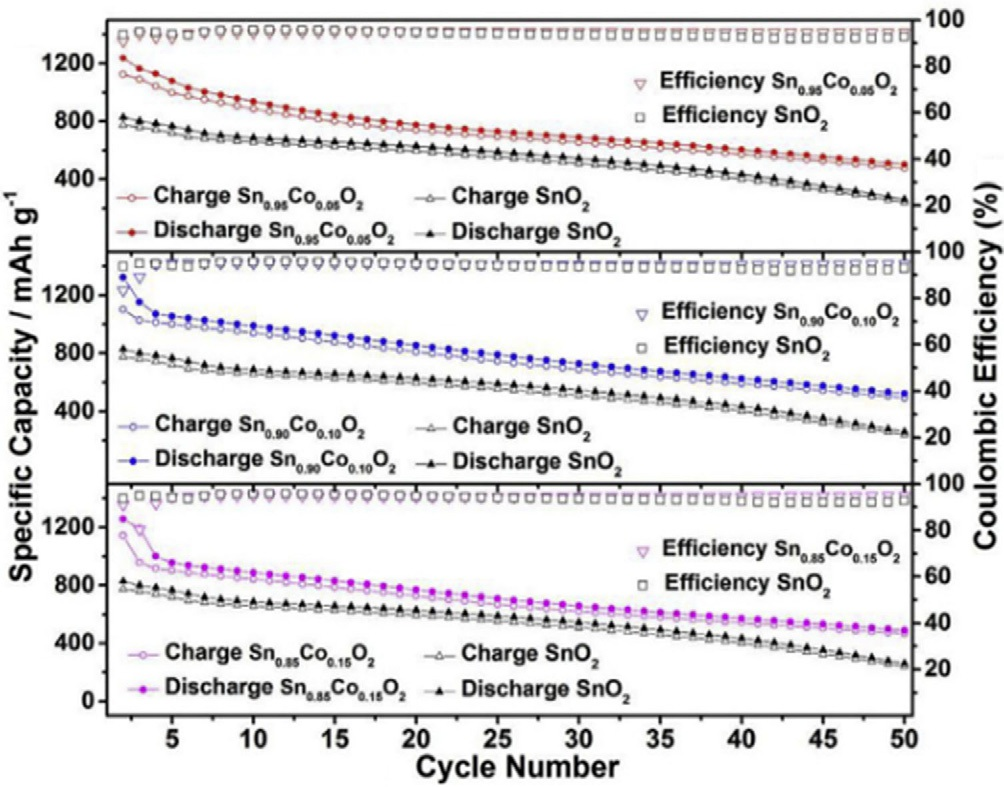
Galvanostatic cycling of Co‐doped SnO_2_‐based anodes. Charge/discharge curves shown for 5, 10, and 15 at % doped SnO_2_ with cycle number. Reproduced with permission from Ref. 4a. Copyright 2018, Elsevier Ltd.

**Figure 12:**
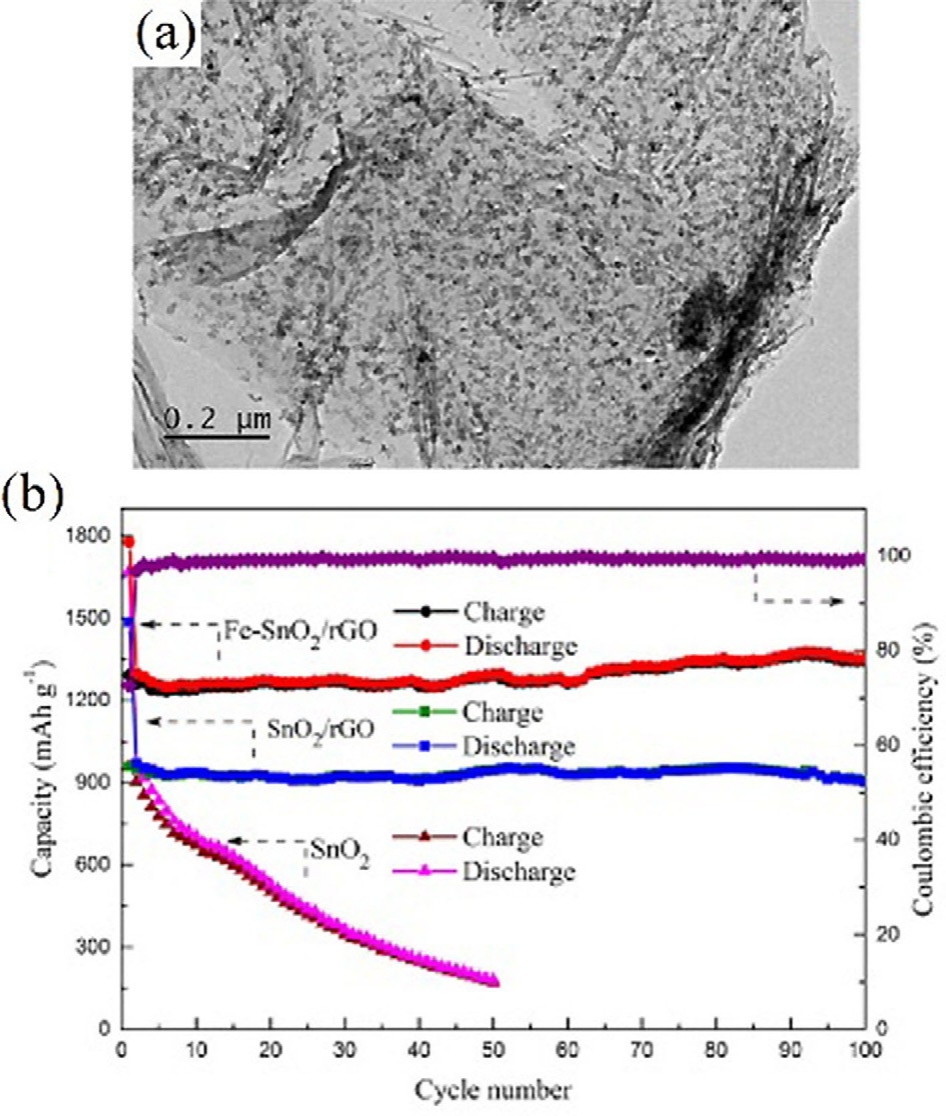
a) TEM image of the Fe−SnO_2_/rGO composite and b) cycling performance of bare SnO_2_, SnO_2_/rGO, and Fe−SnO_2_/rGO electrodes at 0.1 A g^−1^ and the coulombic efficiency of the Fe−SnO_2_/rGO electrode. Reproduced (adapted) with permission from Ref. 53b. Copyright 2018, Elsevier.

Not only does the nature of the dopant, but also the doping ratio, strongly influence the electrochemical performance, as studied by Ma et al., who compared pure SnO_2_ with Co‐doped SnO_2_ with cobalt concentrations of 5, 10, and 15 %.4a They found that the particle size decreased with increasing dopant concentration. A dopant ratio of 10 % (Sn_0.9._Co_0.10_O_2_) provided the best cycling stability of four investigated materials. After 50 cycles at 0.1 A g^−1^, a specific capacity of 493 mAh g^−1^ was obtained for the Sn_0.9_Co_0.10_O_2_ sample, compared with 242, 464, and 476 mAh g^−1^ for SnO_2_, Sn_0.85_Co_0.15_O_2_, and Sn_0.95_Co_0.05_O_2_, respectively (Figure 11). Moreover, Ma et al. also demonstrated that the electrochemical performance of Sn_0.9_Co_0.10_O_2_ could be further enhanced by carbon coating.4a The influence of carbon and its derivatives on the electrochemical performance of SnO_2_/C composites is reviewed in more detail in Section 4. Very promising results regarding the incorporation of transition metals into SnO_2_ were also reported by Wang et al.53b The authors compared the electrochemical performance of an Fe‐doped SnO_2_/reduced graphene oxide (rGO) nanocomposite with undoped SnO_2_/rGO and pristine SnO_2_ nanoparticles; all of them synthesized through a wet chemical approach. TEM measurements showed that the 6–8 nm small SnO_2_ and Fe−SnO_2_ nanoparticles were highly dispersed (Figure 12) over the rGO sheets; this is beneficial for buffering volume changes upon cycling (see Section 4.3), and hence, influences the cycling performance: the bare SnO_2_ electrode reached only 172 mAh g^−1^ after 60 cycles at 0.1 A g^−1^ compared with 905 mAh g^−1^ for the rGO composite after 100 cycles (Figure 12). The Fe−SnO_2_/rGO nanocomposite even retained a capacity of 1353 mAh g^−1^ after 100 cycles. The performance improvement is attributed to iron doping because it leads to better electrical conductivity and encourages the conversion reaction. Consequently, the rate performance of the Fe−SnO_2_/rGO nanocomposite is also superior to that of the undoped analogue.53b

## 4 SnO_2_‐Based Composite LIB Anodes

The use of SnO_2_ together with a carbonaceous material has positive effects on the electrochemical performance.4a The carbonaceous support can buffer volume changes that occur during the alloying/dealloying processes, suppress pulverization and agglomeration of the electrode material, and enhance the overall electrical conductivity in the material.18c, 18f SnO_2_/carbon composites are synthesized either from SnO_2_ active material together with a molecular organic carbon precursor or from preformed carbon allotrope based precursors. Beyond the use of carbon, various metal‐based components, especially transition‐metal chalcogenides, were investigated for the fabrication of composite anodes with SnO_2_ for superior electrochemical performance.

### 4.1 Amorphous carbon (SnO_2_/C composites)

There are different synthetic routes to obtain an amorphous carbon layer coated on SnO_2_ as an active electrode material. One approach is to use both SnO_2_ and carbonaceous precursors to form SnO_2_ and the carbon layer in situ.18a, 22b, 63 A further synthetic route utilizes preformed 3D carbon structures present during SnO_2_ synthesis.^64^ A third possible strategy is to synthesize SnO_2_ first and subsequently treat it with a carbon precursor.18b–18f, 19b, 65 This is especially helpful for retaining the morphology of SnO_2_ compounds with exceptional structures.

**Figure 13:**
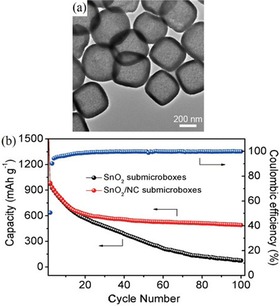
TEM (a) image of SnO_2_/NC sub‐microboxes and their cycling performance at 0.5 A g^−1^ compared to SnO_2_ sub‐microboxes (b). Reproduced (adapted) with permission from Ref. 18c. Copyright 2018, Wiley‐VCH.

Zhou et al., for example, used the last approach to preserve the “sub‐microbox” structure of SnO_2_.18c They used N‐doped carbon, instead of pure carbon, which was supposed to further enhance the conductivity and electrochemical performance. The sub‐microboxes were prepared by means of a multistep synthetic strategy in which Fe_2_O_3_ sub‐microcubes served as templates to be covered with SnO_2_ particles in an in situ hydrothermal process. The resulting core–shell structure was then covered with a smooth layer of polydopamine, which was converted into N‐doped carbon by annealing at 500 °C under nitrogen. Finally, the Fe_2_O_3_ core was removed by etching with oxalic acid. The resulting SnO_2_/N‐doped carbon (SnO_2_/NC) sub‐microboxes have an average size of 400 nm constructed from nanoparticles with sizes of 4–5 nm. Zhou et al. could show that SnO_2_/NC displayed a better cycling performance and rate capability than that of uncoated SnO_2_ sub‐microboxes. After 100 cycles at 0.5 A g^−1^, capacities of 491 and 75 mAh g^−1^ were obtained for the NC‐coated and “pure” SnO_2_ sub‐microboxes, respectively (Figure 13). The authors attributed the superior electrochemical performance of the SnO_2_/NC sub‐microboxes to the large specific surface area and pore volume, small particle size, and increased conductivity supplied by the NC.

### 4.2 CNTs (SnO_2_/C composites)

CNTs are an important example of 1D nanostructured carbon support materials. The use of CNTs together with SnO_2_ can add attractive features. The CNTs can improve the electrical conductivity, buffer volume changes during alloying/dealloying with Li ions, and enable fast electron‐transfer pathways.19c–19e The first step in the synthesis of SnO_2_/CNT composites is often a harsh treatment of pristine CNTs with strong acids or strong oxidizing agents. This creates functional groups on the CNTs that can be used to anchor SnO_2_ particles.19e, 66 Such treatment leads, however, to structural damage and decreased electrical conductivity.19e Ma et al. reported a synthesis without the oxidation of CNTs.19e They used glucose as a mediating agent during hydrothermal synthesis to assist in the in situ formation of 7 nm SnO_2_ particles and serve as a carbon source. The glucose‐assisted SnO_2_/CNT composites exhibited a superior cycling performance. After 150 cycles at 1 A g^−1^, a specific capacity of around 900 mAh g^−1^ was retained, compared with around 450 mAh g^−1^ for the unmediated SnO_2_/CNT composite. Pure SnO_2_ exhibits even lower values. The glucose‐assisted SnO_2_/CNT composites also showed a superior cycling performance at different C rates; this was also attributed to the unique structure and, consequently, enhanced electrical conductivity.19e Cheng et al. reported that the Sn−C bond content played a crucial role.19d They synthesized SnO_2_/CNT composites through a hydrothermal approach by using commercial functionalized multiwalled CNTs followed by an annealing step at different temperatures. The Sn−C fraction strongly depends on this step. The composite annealed at 500 °C exhibited the best cycling and rate performance, compared with those of composites heated at 400 and 600 °C. The first compound demonstrates a capacity of around 600 mAh g^−1^ after 400 cycles at 0.2 A g^−1^, whereas the other two have capacities of only 323 and 211 mAh g^−1^, respectively, after 200 cycles. The authors attributed the promising electrochemical performance to the interplay of the particle size; conductivity; and, most importantly, favorable Sn−C bonding in the SnO_2_/CNT composite.19d

### 4.3 Graphene (SnO_2_/C composites)

**Figure 14:**
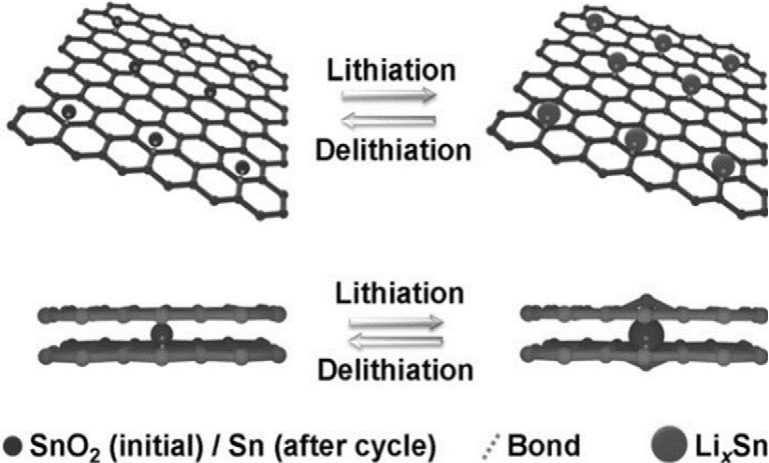
Schematic illustration of lithiation/delithiation processes in a SnO_2_ nanocrystal/graphene composite. Reproduced (adapted) with permission from Ref. 67c. Copyright 2014, Wiley‐VCH.

Graphene is an important 2D carbonaceous support material with exceptional properties, such as very good electrical conductivity, large surface area, high theoretical capacity of 744 mAh g^−1^, and excellent mechanical properties. The last of these, for example, can help to avoid aggregation of SnO_2_ particles and buffer volume changes during alloying/dealloying with Li ions; thus leading to better cycling stability (Figure 14).23a, 67

SnO_2_/graphene composites can be obtained by simply mixing SnO_2_ with graphene or graphene oxide (GO) or through an in situ method, which is more common.23c, 68 For the latter, graphene or GO is treated with a tin precursor (e.g., SnCl_4_ or SnSO_4_) to form SnO_2_ particles attached to the graphene or GO surface. In particular, functional groups such as epoxide, carbonyl, or hydroxyl, which can be found on the GO surface, are attractive anchor points for the tin precursors.23b, 67a, 69 If GO has not been reduced to graphene during the synthesis, there are two popular options: the use of a strong reducing agent (e.g., hydrazine) or heating the sample under a reducing or inert atmosphere, for example, H_2_ or N_2_. The obtained graphene/rGO has a superior conductivity to that of GO.23b, 23c, 70 Zhang et al. showed that this had a positive effect on the electrochemical performance of SnO_2_/graphene composites.23b They used a pH‐dependent, one‐pot hydrothermal method to grow SnO_2_ nanoparticles (2–5 nm) in situ onto the surface of graphene sheets. The SnO_2_/rGO nanocomposite delivers a specific capacity of 942 mAh g^−1^ after 80 cycles at 100 mA g^−1^, compared with 827 and 142 mAh g^−1^ for SnO_2_/GO and pristine SnO_2_ particles, respectively. The SnO_2_/rGO nanocomposite also exhibits a superior rate capability.23b

However, SnO_2_ particles can aggregate on graphene sheets during cyclic lithiation/delithiation reactions, which could lead to a loss in capacity.^69^ Carbon coating of SnO_2_ particles can avoid the formation of such agglomerates, as discussed previously herein. Hence, the use of both carbon coating and graphene as a support is reported to be advantageous for the electrochemical performance. For example, Zhang et al. presented a carbon‐coated SnO_2_ graphene (rGO/PC/SnO_2_) nanocomposite with an improved rate performance and cycling stability to that of an uncoated reference composite.^69^ The SnO_2_ nanoparticles are formed in situ on the GO sheets through a solvothermal approach, with a size of around 4 nm. The additionally added glucose served both as a soft template and as a carbon‐coating source. The rGO/PC/SnO_2_ nanocomposite exhibits a capacity of 1468 mAh g^−1^ after 150 cycles at 0.1 C, relative to 200 mAh g^−1^ for the uncoated sample. The rate performance of the coated nanocomposite is also superior. The authors argued that this excellent performance was caused by the small particle size, good conductivity, large electrolyte–active material interface, and mechanical stabilization of the nanocomposite.

Importantly, not only SnO_2_, but also graphene sheets, can suffer from some kind of aggregation. Graphene sheets tend to restack due to π–π interactions, which implies an inferior compensation of the volume changes of SnO_2_ and, as a consequence, a reduced electrochemical performance.1b, 23a Fabrication of 3D structures and/or the introduction of a buffering layer are reported to prevent the restacking of individual graphene sheets, which has positive effects on the electrochemical performance.23a, 71 The 3D graphene structures, such as graphene foams, aerogels, or skeletons, can have an increased surface area and more voids to host and/or encapsulate SnO_2_ particles. The latter can be beneficial to alleviate volume changes; hence increasing the structural stability and electrochemical performance of SnO_2_/graphene composites.^71^, ^72^ Liu et al., for example, used a spray‐drying approach to prepare a SnO_2_/skeleton‐structured 3D network of graphene sheets.^71^ Their composite exhibits a specific capacity of 1140 mAh g^−1^ after 120 cycles, relative to 121 mAh g^−1^ after 50 cycles for pristine SnO_2_ (at 100 mA g^−1^). They attributed the improved electrochemical performance to the skeleton‐like 3D structure, which could buffer the volume changes of SnO_2_ and was beneficial for electrolyte transport and the diffusion of lithium ions.

**Figure 15:**
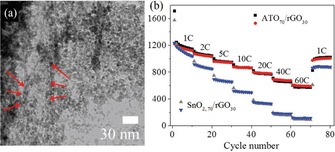
a) HR‐TEM image of Sb:SnO_2_ nanoparticles on rGO sheets. b) Specific capacity of galvanostatic charge/discharge measurements at C rates up to 60 C. Reproduced with permission from Ref. 44b. Copyright 2018, Wiley‐VCH.

Another strategy to improve the performance of SnO_2_‐based anode materials is to use doped SnO_2_ nanoparticles and graphene as a support material.44b, 48, 50, 53b, 54b, 55, 56, 59b–59d Zoller et al. demonstrated that the electrochemical performance of Sb‐doped SnO_2_ (ATO)/rGO composite was superior to that of SnO_2_/rGO and unsupported ATO particles. The composites and pure ATO were synthesized through a microwave‐assisted solvothermal approach, which led to SnO_2_ and ATO particles of around 3–4 nm in size. The superior electrochemical performance of the ATO/rGO composite, relative to those of SnO_2_/rGO and pure ATO, was especially demonstrated in performance tests at high C rates of up to 60 C (Figure 15).44b

Additionally, graphene can be functionalized and doped with nitrogen8c, 73 and/or sulfur,^74^ which can further enhance the electrochemical rate performance of the SnO_2_/graphene composites, as demonstrated in the recent work by Wu et al.74a The authors showed that SnO_2_ quantum dots anchored on sulfur‐doped rGO (S‐rGO) outperformed the analogous undoped rGO composite in terms of rate capability and cycling stability; this was attributed to sulfur doping of graphene resulting in an improved structural stability and better charge and ion conduction at the electrode interface.

### 4.4 SnO_2_/non‐carbonaceous composites

Much research has been conducted in the field of composite materials of SnO_2_ together with metal oxides and sulfides, such as CoS,^75^ SnS,^76^ SnS_2_,^77^ MoS_2,_ 78 CoO,^79^ Co_3_O_4_,^80^ CuO,^81^ Fe_2_O_3_,^82^ MnO_2_,^83^ Mn_2_O_3_,^84^ MoO_3_,^85^ NiO,^86^ WO_3_,^87^ TiO_2_,^88^ Li_4_Ti_5_O_12_ (LTO),^89^ VO_2_,^90^ SiO_2_,^91^ or ZnO.^92^ Additionally, SnO_2_/C_3_N_4_ 93 and SnO_2_/titanium carbide nanosheets (MXene)^94^ are among reported hybrid materials. The SnO_2_ composites are often additionally supported by carbonaceous matrices. In general, the improved electrochemical performance of these composites compared with the phase‐pure counterparts is attributed to synergistic effects between the components.

In the case of SnO_2_/metal sulfide (M_*x*_S_*y*_; M=Sn, Mo) composites, the individual compounds have different band gap energies that enable the formation of heterojunctions.^77^, 78b, 95 As mentioned in Section 3, SnO_2_ is a wide band gap (3.8 eV) n‐type semiconductor, whereas SnS is a narrow‐band‐gap (1.3 eV) p‐type semiconductor, for example.^76^ A p–n heterojunction forms at the interface between SnO_2_ and the metal sulfide. This entails holes diffusing from the metal sulfide to SnO_2_ and electrons diffusing in the opposing direction; thus leading to the formation of a depletion region and the formation of an internal electric field. This enhances charge‐transfer kinetics through increased carrier mobilities and thereby eventually results in a higher conductivity.^76^, ^96^

**Figure 16:**
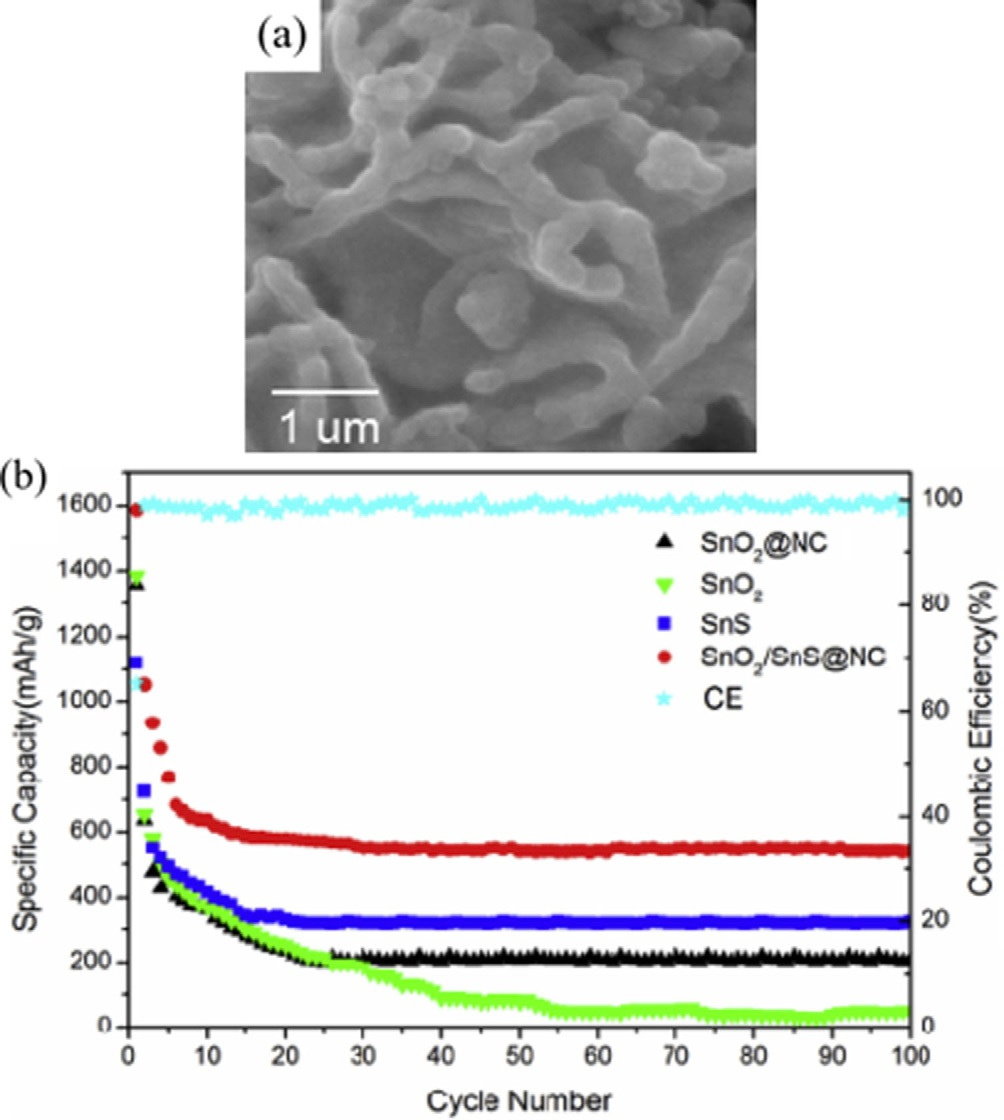
a) SEM image of the SnO_2_/SnS/NC nanocomposite and b) its cycling performance compared with those of SnS, SnO_2_, and SnO_2_/NC. Reproduced (adapted) with permission from Ref. 76a. Copyright 2018, Elsevier B.V.

In this context, Ye et al. demonstrated that SnO_2_/SnS NC composite showed a superior electrochemical performance to those of pure SnS, SnO_2_, and SnO_2_/NC, reaching values of 550, 300, 200, and 50 mAh g^−1^, respectively, after 100 cycles at 0.1 A g^−1^ (Figure 16).76a The authors also demonstrated an improved rate performance for the SnO_2_/SnS/NC nanocomposite; thus underlining the beneficial effect of the formation of the SnO_2_/SnS heterojunction on the conductivity of the active material.

However, the improved electrochemical performance of SnO_2_/metal oxide (M_*x*_O_*y*_; M=Co, Cu, Fe, Mn, Mo, Ni, W, etc.) hybrids compared with that of SnO_2_ is associated with sequential lithiation at different potentials of SnO_2_ and M_*x*_O_*y*_.81b, 82a, 82d, 85, 86b, 87b Consequently, if the SnO_2_ nanoparticles are reduced, at the same time, the M_*x*_O_*y*_ particles are practically electrochemically inactive and can buffer volume changes and prevent newly formed Sn particles from aggregating.82d Additionally, it was reported that, upon cycling, in situ generated metal nanoparticles from the M_*x*_O_*y*_ phase catalytically decomposed the formed Li_2_O matrix, which increased the overall capacity and cycling stability.^79^, ^80^, 81b, 82a, 84b, 86b, 87b

Notably, titanium oxides in SnO_2_/M_*x*_O_*y*_ composites are “zero” or low‐strain materials that display negligible volume changes upon lithiation/delithiation, with the downside of a low specific capacity. Titanium oxides can therefore be used to preserve the nanostructure of SnO_2_ by physical confinement and anchoring.88c, 88d, 97

The class of 2D metal carbides and nitrides known as MXene has gained considerable attention for composite formation in recent years.^98^ The synergistic effect in SnO_2_/MXene anodes is based, on one hand, on the very good electronic conductivity and enhanced lithium‐ion transport ability of the layered MXene structures, together with their mechanical flexibility, which is important for buffering the volume changes of SnO_2_. On the other hand, SnO_2_ prevents the MXene sheets from restacking, and thus, improves the cyclability remarkably.^94^

**Figure 17:**
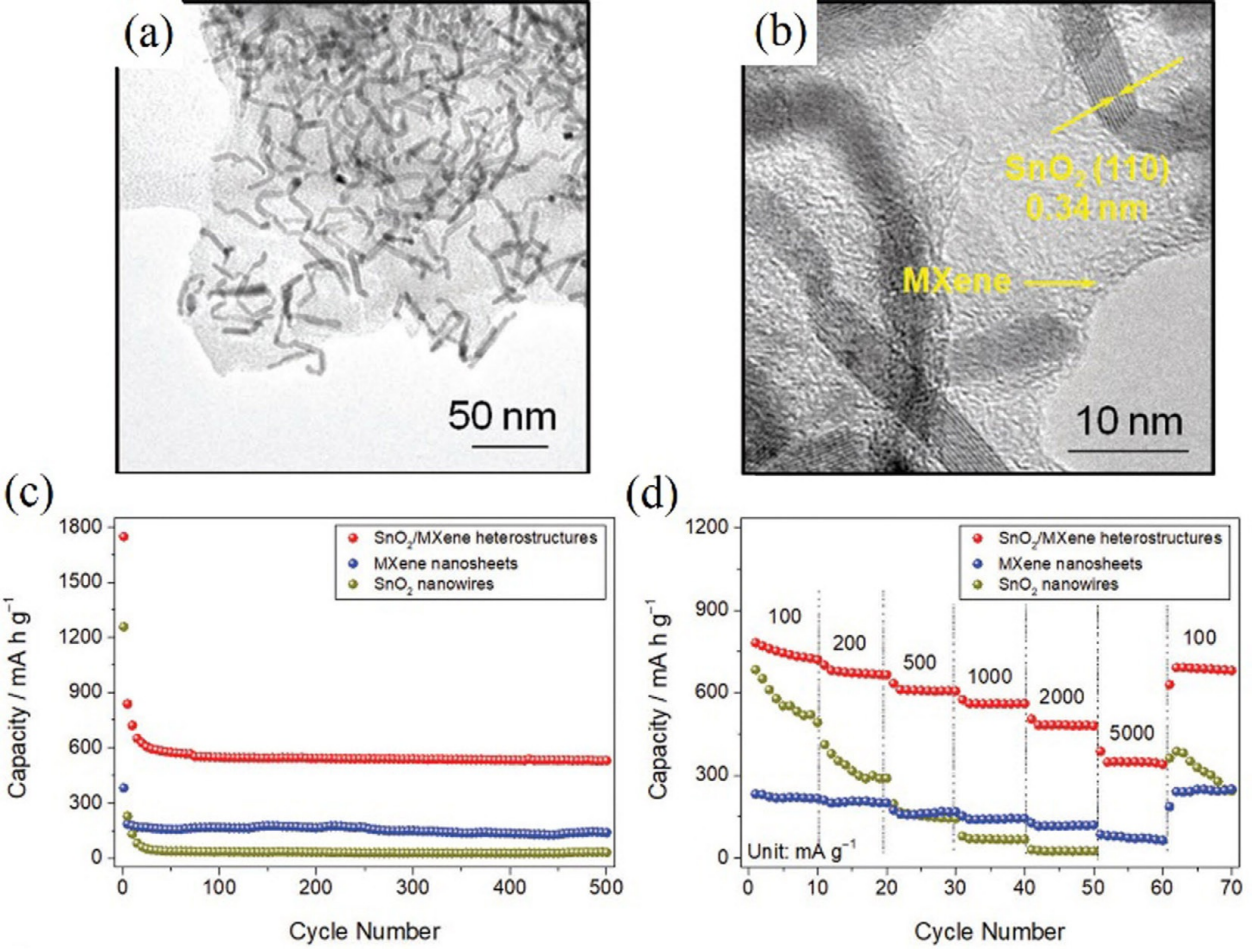
a) High‐magnification TEM and b) HRTEM images of a SnO_2_ nanowire/Ti_3_C_2_(MXene nanosheet) nanocomposite. c) Cycling performance at 1 A g^−1^ and d) rate capability at different current densities of this composite, in comparison with the phase‐pure counterparts. Reproduced (adapted) with permission from Ref. 94a. Copyright 2018, Wiley‐VCH.

This was, for example, successfully demonstrated by Liu et al.94a They compared the cycling performance of a SnO_2_ nanowire/Ti_3_C_2_(MXene) nanosheet composite, SnO_2_ nanowires, and Ti_3_C_2_ (MXene) nanosheets (Figure 17), and obtained values of 530, 31, and 139 mAh g^−1^, respectively, after 500 cycles at 1 A g^−1^. The rate performance measurements also confirmed the improved electrochemical performance of the SnO_2_ nanowire/MXene composite.

A further example of a SnO_2_/non‐carbonaceous composite was presented by Idota et al., who embedded redox‐active Sn^II^ centers into an amorphous glass‐forming matrix of −(M−O)− elements composed of B^III^, P^V^, and Al^III^, resulting in an amorphous SnM_*x*_O_*y*_ composite.^3^ A reversible capacity of >600 mAh g^−1^ was reported at a charge/discharge current of 48 mA g^−1^, with a capacity retention of >90 % after 100 cycles in a full‐cell configuration with a LiCoO_2_ (LCO) cathode.

## 5 Full LIB Cell Performance with SnO_2_‐Based Anodes

Because SnO_2_‐based materials exhibit very promising results in half‐cells (meaning with Na or Li metal foil as the cathode), there is growing interest in testing these materials in full cells to evaluate their possible application in LIBs. Mismatching charge/discharge potentials and kinetics of corresponding anode–cathode materials may result in low performance and/or fast degradation of the active material.^99^ Table 1 presents an overview of performance data for full‐cell assemblies employing SnO_2_‐based anodes and the most commonly used lithium cobalt oxide based cathode materials.

**Table 1:** Overview of lithium‐ion full‐cell battery capacities with SnO_2_‐based anodes.

Anode	Cathode	Capacity [mAh g^−1^] (cycle no.), potential window [V]	Current density [A g^−1^]	Ref.
SnO_2_/N,S codoped graphene	LCO	356.4 (100), 1.2–3.9	0.1	100
S/F‐doped SnO_2_/GO	Li[Ni_0.6_Co_0.2_Mn_0.2_]O_2_ (NCM)	≈25^[a]^	0.01	62
SnO_2_/C/graphene	LCO	345.8 (90), 1.2–4.2	0.1	101
SnSe/SnO_2_/graphene	LCO	312 (50), 1.0–3.8	0.1	95
SnO_2_−Fe_2_O_3_−C	NCM	≈490 (20), 1.8–4.2	0.1	82a
Zn‐doped SnO_2_/rGO	LiFePO_4_ (LFP)	can light a green/red LED		56
SnO_2_/3D rGO	LCO	≈300 (100), 1.8–4.2	≈0.12 (0.2 C)	102
pretreated SnO_2_^[b]^	Li_0.995_V_0.005_Ni_0.5_Mn_1.5_O_4_ (LVNMO)	≈475 (50), 3.7–4.7	0.1	35
SnO_2_/NC/TiO_2_	LFP	135 (50), 2.0–4.0	0.1	88d

[a] Original electrode area‐based value: 2.7 mAh cm^−2^ (10). [b] Pretreatment of the anode by two full discharge/charge cycles to eliminate ICL in the full cell.

**Figure 18:**
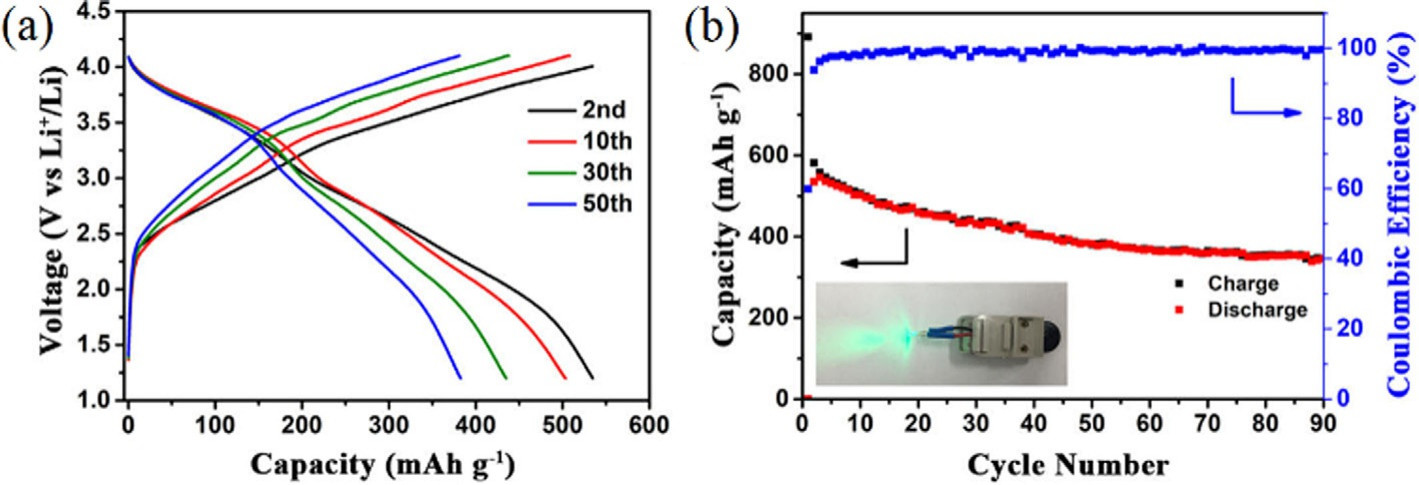
Full‐cell LIBs with SnO_2_/C/graphene composite as an anode and commercial LCO as a cathode (based on anode mass). a) Discharge/charge curves, b) cycling performance at 0.1 A g^−1^; inset: a light‐emitting diode (LED) powered by such a full cell. Reproduced (adapted) with permission from Ref. 101. Copyright 2018, American Chemical Society.

Wu et al. reported a composite consisting of hollow SnO_2_ nanospheres, NC, and rGO sheets.^101^ This unique structure enabled an encouraging electrochemical performance, also on the full‐cell level, with commercial LCO as the cathode material (Figure 18). The full cells were investigated over a potential range of 1.2–4.2 V. After 90 cycles at 0.1 A g^−1^, a discharge capacity of 346 mAh g^−1^ (based on the weight of the anode) was reported; this equaled a capacity retention of approximately 67 %.

## 6 SnO_2_‐Based Anodes for NIBs and KIBs

### 6.1 SnO_2_‐based NIB anodes

Since the first successful demonstration of SnO_2_ as a promising anode material in LIBs, there has been growing interest in the use of tin‐based anode materials in NIBs and KIBs. The sodiation reactions of SnO_2_ are similar to those of lithiation and can be described by Equations (5), (6), resulting in a total theoretical specific capacity of 1398 mAh g^−1^:^2^

(5) $$\mathrm{conversion}:\mathrm{SnO}{}_{2}+4\;\mathrm{Na}{}^{+}+4\;e{}^{-}\to \mathrm{Sn}+2\;\mathrm{Na}{}_{2}O$$

(6) $$\mathrm{alloying}/\mathrm{dealloying}:\mathrm{Sn}+x\;\mathrm{Na}{}^{+}+x\;e{}^{-}\to \mathrm{Na}{}_{x}\mathrm{Sn}\;\;\left(0\leq x\leq 3.75\right)$$

The larger ionic diameters of Na^+^ and K^+^ (K^+^>Na^+^>Li^+^; 1.38 Å>1.02 Å>0.76 Å, respectively), however, aggravate problems caused by volume changes upon charge/discharge, and result in a decreased cycling performance compared with that of Li^+^.^101^, ^103^ To tackle these problems, strategies successfully employed for SnO_2_‐based anodes in LIBs, such as nanosizing, 3D structuring, or the introduction of carbonaceous support materials, were also suggested to improve the electrochemical performance in KIBs and NIBs.^2^, ^103^, ^104^

**Figure 19:**
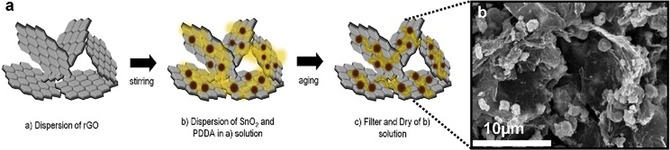
a) Schematic illustration of the synthetic process for the SnO_2_‐nanoparticle/rGO composite and b) a SEM image of the product. Reproduced (adapted) with permission from Ref. 104b. Copyright 2017, Elsevier.

The use of SnO_2_ together with rGO is an example of this development. Jo et al. synthesized a SnO_2_/rGO composite that exhibited an improved electrochemical performance to that of bare SnO_2_ anodes.104b In their approach, SnO_2_ particles were first solvothermally prepared and then attached to the rGO sheets through a layer‐by‐layer self‐assembly process (Figure 19). Cycling tests at 0.1 A g^−1^ revealed capacities of 492 mAh g^−1^ (capacity retention: 80.2 % relative to that of the first charging cycle) for the composite and 194 mAh g^−1^ (42.5 % retention of the initial charge capacity) for SnO_2_ after 100 cycles. The rate performance of the SnO_2_/rGO composite could also be significantly increased from about 250 to 425 mAh g^−1^ at 2.4 A g^−1^ compared with that of bare SnO_2_.104b For the construction of a high energy density sodium ion full cell, they further paired the SnO_2_‐nanoparticle/rGO anode with a C‐NaCrO_2_ cathode. The resulting NaCrO_2_//SnO_2_/rGO composite full cells showed an excellent cycling stability at a rate of 0.5 C (55 mA g^−1^), with a capacity retention of 84 % after 300 cycles and high rate capability tested up to 10 C (87 mAh g^−1^ based on the cathode mass at 1.1 A g^−1^).104b

A further example of a sodium‐ion full‐cell assembly was reported by Lee et al.^102^ In their work, a SnO_2_/3D graphene composite prepared through a hydrothermal approach was paired with self‐produced Na_3_V_2_(PO_4_)_3_ (NVP) serving as a cathode. The anode material was preactivated before the first cycle to avoid alkaline ion consumption during SEI formation. In the case of the SnO_2_/3D graphene‐NVP full cells, a specific capacity of 71 mAh g^−1^ (based on the weight of the anode) was reached after 100 cycles at a rate of 0.05 C.

A further increase in performance was achieved by Wang et al., who used a layer‐by‐layer assembly technique with a porphyrin derivative as an interfacial linker to homogeneously attach SnO_2_ crystals about 5 nm in size onto N and S codoped graphene.^100^ By combining it with a NVP/C cathode, a remarkable full‐cell capacity of 108.2 mAh g^−1^ was measured after 100 cycles at a rate of 0.1 A g^−1^.

Table 2 gives a brief overview of recently published sodium‐ion full‐cell battery performance data.

**Table 2:** Overview of sodium‐ion full‐cell battery capacities with SnO_2_‐based anodes.

Anode	Cathode	Capacity [mAh g^−1^] (cycle no.), potential window [V]	Current density [A g^−1^]	Ref.
SnO_2_/N,S codoped graphene	NVP/C	108.2 (100), 1.0–3.9	0.1	100
SnO_2_/3D rGO	NVP	71 (100), 2.5–3.8	0.055 (0.5 C)	102
SnO_2_/rGO	NaCrO_2_	92 (300), 1.5–3.4	0.055 (0.5 C)	104b

### 6.2 SnO_2_‐based KIB anodes

Inspired by a study on K−Sn alloying and intercalation by Sultana et al.,^105^ Wang et al. published an in situ TEM and diffraction study on the potassiation of Sn nanoparticles in KIBs.^106^ They observed a high volume expansion of about 197 % after an uptake of only one equivalent of K, with the formation of a KSn phase identified by electron diffraction, accompanied by the reversible formation of nanopores and finally pulverization of the active material.^106^ However, in a follow‐up study by Ji et al., on dual‐ion batteries, with Sn foil as the anode, a higher potassium uptake could be observed by means of ex situ XRD measurements, with the formation of a K_2_Sn phase as a final alloying product.^107^

**Figure 20:**
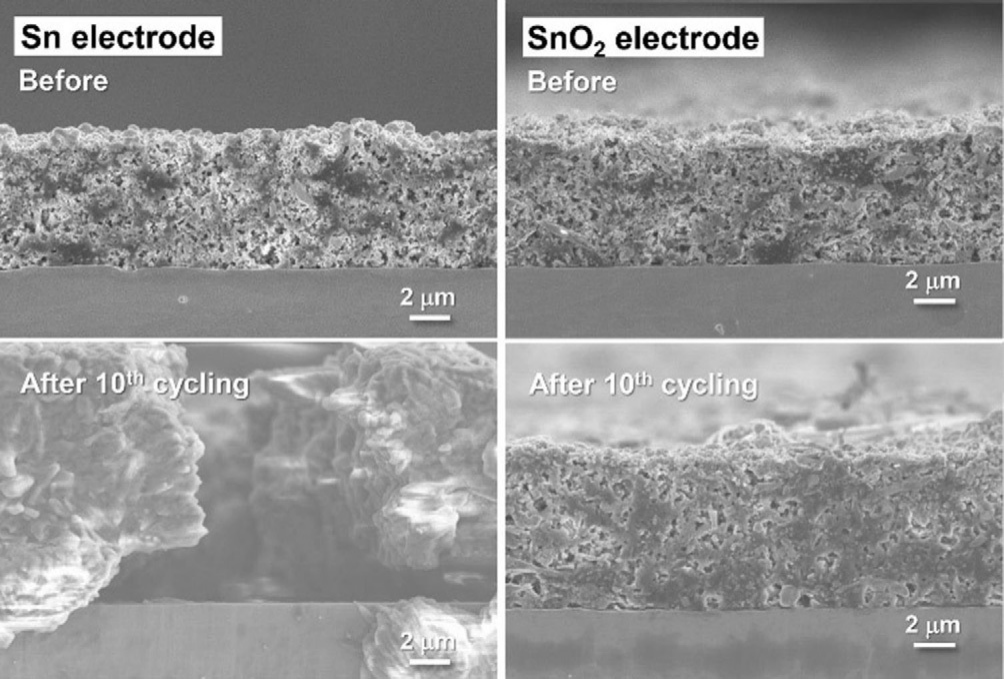
Cross‐sectional field‐emission (FE) SEM images of Sn‐based anodes in KIB half‐cells before and after the 10th cycle under a constant current density of 25 mA g^−1^. Reproduced (adapted) with permission from Ref. 109. Copyright 2018, The American Chemical Society.

Large volume changes induced by the potassiation of metallic Sn and accompanying capacity fading caused by electrode pulverization constitute significant challenges for its application as an anode material in KIBs. However, it has been demonstrated that the use of SnO_2_‐based electrodes, instead of Sn, can significantly mitigate these effects. Similar to lithiation processes, the K_2_O matrix formed in the conversion reactions and surrounding the newly formed Sn (nano) particles can buffer volume changes upon alloying and suppress aggregation.^108^ The positive influence of the K_2_O matrix formed around Sn nanoparticles on the structural integrity of the tin oxide based anodes for KIBs, in contrast to metallic Sn‐based electrodes, was demonstrated, for example, by Shimizu et al. (Figure 20).^109^ They precipitated SnCl_2_ precursor, with subsequent thermal oxidation, to obtain a 10 μm sized flowerlike morphology composed of SnO_2_ sheets of about 100 nm as primary building blocks. The resulting electrodes exhibit a rather limited potassium storage capability of about 25 mAh g^−1^ at a rate of 0.025 A g^−1^, but demonstrate stability over 50 cycles.^109^

Huang et al. recently investigated the potassium‐storage capability of SnO_2_–carbon nanofibers synthesized by means of electrospinning of a precursor solution containing SnCl_2_/polyacrylonitrile (PAN)/polymethylmethacrylate (PMMA), with a subsequent pyrolysis step, to obtain fibers with a diameter of about 490 nm and several micrometers in length.104a The focus of their work was on enhancing electrode conductivity by the addition of graphene to the electrospinning process and a synergistic effect on the K^+^ storage behavior among the SnO_2_, rGO, and carbon constituents. As a result, the capacity could be increased from about 170 (SnO_2_/C) to 250 mAh g^−1^ (SnO_2_/rGO/C) upon cycling at a rate of 0.1 A g^−1^.104a

In a follow‐up paper by Huang et al., P doping of SnO_2_/rGO/C by phosphoric acid was reported, with the aim of further increasing the electrochemical performance.^103^ The electrospinning process of a GO/(H_3_PO_4_)/SnCl_4_/PVP‐containing precursor solution yielded nanofibers of about 150 (non‐P‐doped) and 120 nm in diameter (P‐doped) and micrometers in length. The cycling performance at a rate of 0.1 A g^−1^ could be increased from about 206 (undoped material) to 285 mAh g^−1^ (P‐doped), both determined for the 60th cycle. The authors hypothesize that modification with H_3_PO_4_ had several beneficial effects on the K^+^ diffusion kinetics. These include the formation of a beneficial mesoporous structure, an increase in conductivity, and a widening of the interlayer spacing of rGO, which is reflected in a reversible capacity of 200 mAh g^−1^ at a high rate of 1 A g^−1^.^103^

**Figure 21:**
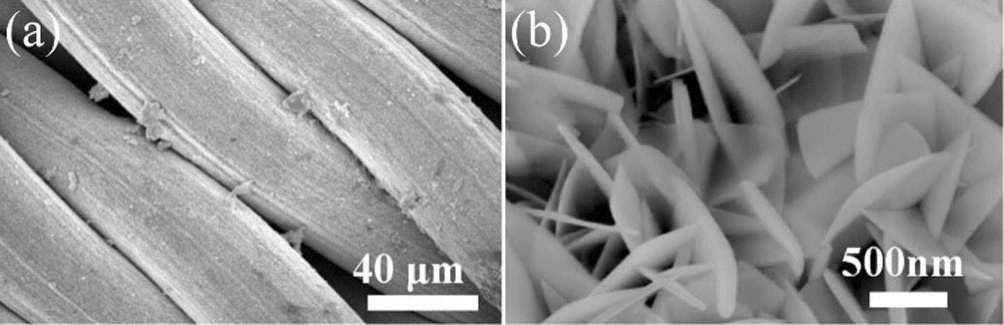
SEM images of SnO_2_ nanosheets (b) synthesized on a SSM electrode (a). Reproduced (adapted) with permission from Ref. 108. Copyright 2018, Elsevier.

The best performing SnO_2_ anode for KIBs so far, to the best of our knowledge, was recently published by Suo et al., who prepared a binder‐free SnO_2_‐nanosheet/stainless‐steel mesh (SSM) anode through solvothermal synthesis with a SnCl_2_ precursor in the presence of the mesh (Figure 21).^108^ An initial discharge capacity of 603 mAh g^−1^ was determined for this material, which stabilized within 5 cycles at a reversible capacity of about 450 mAh g^−1^. Within 100 cycles, a moderate decrease in capacity to 339 mAh g^−1^ was observed. The prepared anode material also showed a good rate capability of 125 mAh g^−1^ at 1 A g^−1^.

Table 3 presents an overview of SnO_2_‐based anode materials for application in KIBs tested in half‐cell configurations.

**Table 3:** Overview of the electrochemical storage properties of SnO_2_‐based KIB anodes (half‐cell measurements).

Anode	Capacity [mAh g^−1^] (cycle no.), potential window [V]	Current density [A g^−1^]	Ref.
SnO_2_‐carbon nanofibers (SC) (≈490 nm Ø)	≈170 (60), 0–2.5	0.1	104a
SnO_2_‐rGO‐carbon nanofibers (SGC) (≈490 nm Ø)	≈250 (60), 0–2.5	0.1	104a
SGC nanofibers (≈150 nm Ø)	206 (60), 0.001–3.0	0.1	103
phosphoric acid doped SnO_2_‐rGO‐carbon (P‐SGC) nanofibers (≈120 nm Ø)	285 (60), 0.001–3.0	0.1	103
SnO_2_ nanosheets on SSM (binder‐free)	351 (100), 0.02–2.6	0.05	108

## 7 Summary and Outlook

The alloying of alkali ions with tin results in a high theoretical volumetric and gravimetric charge capacity, which is accompanied by volume changes of up to 200^106^–250 %5c (for K^+^ and Li^+^, respectively). Large volume changes pose a major challenge for the mechanical and structural integrity of the electrode upon cycling.5c, 106 To address this problem, much effort was dedicated to fabricate diverse 0D–3D SnO_2_ nanostructures. Based on an analysis of the most recent developments, herein, we aimed to elucidate the relationship between the nanostructure, synthetic route employed (resulting phase), and the electrochemical performance of phase‐pure SnO_2_. It can be concluded that the optimum size of SnO_2_ nanocrystals, with respect to reversible capacity and cyclability, strongly depends on the exact nature (crystallinity and dominating crystal facets determined by the synthetic conditions) and spatial distribution of nanosized Sn and its surrounding amorphous Li_2_O matrix formed during the initial conversion reaction. From the performance data of recently published articles with differing SnO_2_ nanomorphologies and crystallite sizes, we conclude that particles with a size smaller than 10 nm may yield anodes with a high ion‐storage capacity and reversibility,11b which, however, cannot effectively be enhanced by nanostructuring.

As another means to improve the electrochemical performance of SnO_2_ anodes, doping with either redox‐active or ‐inactive atoms was explored by many research groups. We conclude that the increase in electrochemical performance (capacity and rate) observed is associated with an increase in conductivity (known for Sb)44a induced by a modification of the band structure of the wide band semiconductor SnO_2_. Additionally, among a variety of investigated transition metals, cobalt is very promising because Co‐doped SnO_2_ was also reported to show a volume buffering effect, which might additionally increase its cyclability.4a

On the electrode level, carbon composite formation in the form of SnO_2_/(doped)graphene, SnO_2_/CNT, SnO_2_/amorphous carbon, and/or their combination was discussed as a very efficient strategy to improve the anode performance, in terms of storage capacity and cyclability. Graphene‐type carbon (undoped rGO44b or doped with N,8c, 73 S,^74^ or P),^103^ with a high surface area and high conductivity, is often used as a support for the homogeneous attachment of nanosized SnO_2_‐based active materials. Together with a thin layer of amorphous carbon obtained through the pyrolysis of organic molecules in the precursor mixture, this results in a highly conductive, flexible, and porous matrix.23b, 23c, 44b The best performing composite anodes with transition‐metal‐doped nanostructured SnO_2_ showed a remarkable reversible capacity of over 1200 mAh g^−1^ (after 100 cycles at 0.1 A g^−1^),94a which greatly outperformed that of standard graphite anodes (e.g., ≈226 mAh g^−1^ cycled at 0.5 C for 100 cycles with a loading of 10.1 mg cm^−2^)^110^ in classical LIBs by more than a factor of five. However, regarding the future commercialization of SnO_2_‐based anodes, two objectives need to be addressed. First, high‐capacity and rate‐capable anodes, with mass loadings in the range of 10 mg cm^−2^_,_ 111 need to be realized. Second, and most important, for practical applications is the combination with a suitable high‐rate‐capable, high‐voltage cathode material to obtain full cells with equal or increased energy density to that of classical LIBs employing only carbonaceous anodes.

Future work could include the combination of SnO_2_‐based anodes with high‐voltage cathodes, exceeding the stability window of conventional carbonate electrolytes (EC, ethyl methyl carbonate, diethyl carbonate, etc.), which would require the use of respective additives or ionic‐liquid‐based electrolytes.^112^

From the perspective of increased operational safety, which is already increased at the anode side by the replacement of graphitic carbon with SnO_2_, a solid electrolyte that allows for a high‐voltage window (e.g., NaSICON‐type or LiGe_2_(PO_4_)_3_‐type)^112^ would be beneficial. The high rate capability and increased gravimetric capacity, relative to that of graphite electrodes, paired with increased operational safety renders SnO_2_‐based anodes interesting for applications in future energy‐storage devices in the industrial and automotive sector.

NIBs with SnO_2_‐based anodes have gained considerable attention in recent years, with the first published examples of full cells. Knowledge transfer from the design of LIBs resulted in the fabrication of full cells with reversible capacities of up to about 108 mAh g^−1^ after 100 cycles at 0.1 C.^100^ It can be expected that research into SnO_2_‐based anodes for NIBs will intensify due to the general attractiveness of NIBs, such as low cost, high abundance of sodium, low toxicity, and increased safety due to a lack of dendrite formation.

Although research into KIBs with SnO_2_‐based anodes is very new, rapid progress has been made due to knowledge transfer (synthesis of active materials, anode architecture, and methodology) from LIBs and NIBs with SnO_2_‐based anodes. However, the processes taking place during reversible potassiation/depotassiation of tin and occurring intermediate phases^2^ still have to be clarified, although the first publications have identified possible K−Sn alloys.^106^, ^109^ Fabricated KIB half‐cells have shown a capacity of up to 351 mAh g^−1^ for a pure, binder‐free SnO_2_ nanosheet anode,^108^ and results for the first full cells are expected in the near future.

## Conflict of interest

*The authors declare no conflict of interest*.

## Biographical Information

Florian Zoller is a Ph.D. student in the Fattakhova‐Rohlfing group at the Universität Duisburg‐Essen (UDE). He received his BSc. degree in chemistry and biochemistry and his M.Sc. degree in chemistry from Ludwig‐Maximilians‐Universität München (LMU). His current research interests include novel nanostructured lithium‐ion battery anode and cathode materials as well as electrocatalysis.

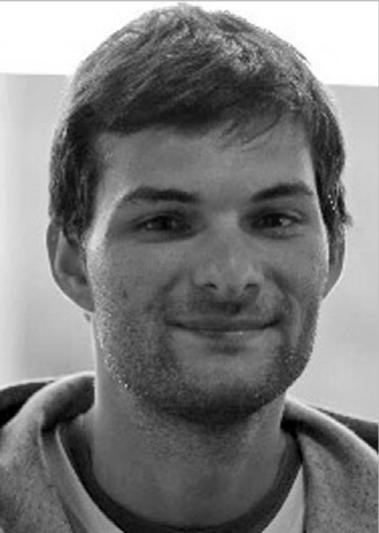

## Biographical Information

Daniel Böhm is a Ph.D. student in the Fattakhova‐Rohlfing group at the Ludwig‐Maximilians‐Universität München (LMU). He received his B.Sc. degree in chemistry and biochemistry and his M.Sc. degree in chemistry from the LMU. His current research interests include novel nanostructured lithium‐ion battery anode materials and electrocatalysis for water‐splitting applications.

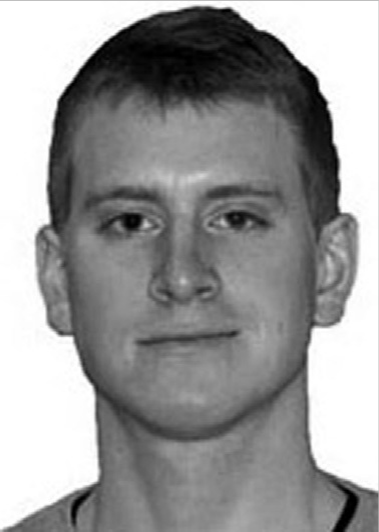

## Biographical Information

Thomas Bein is Chair of Physical Chemistry at the Ludwig‐Maximilians‐Universität München (LMU). He leads a research team dedicated to the discovery and translation of novel functional nanostructures related to renewable energy conversion technologies and biomedical applications.

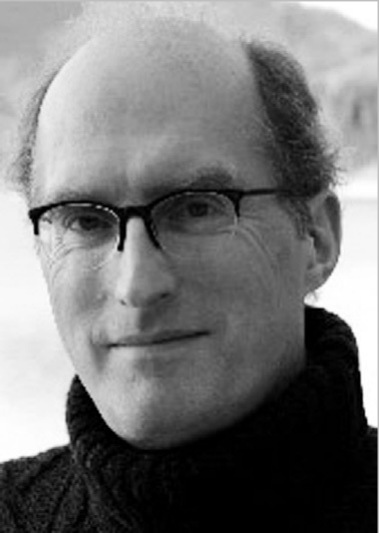

## Biographical Information

Dina Fattakhova‐Rohlfing is Head of the Department of Electrochemical Storage at the Institute of Energy and Climate research (IEK‐1) at Forschungszentrum Jülich (FZJ) and Professor at the Universität Duisburg‐Essen (UDE). Her research is focused on the development of materials for electrochemical applications, including electrocatalysis and electrochemical energy storage.

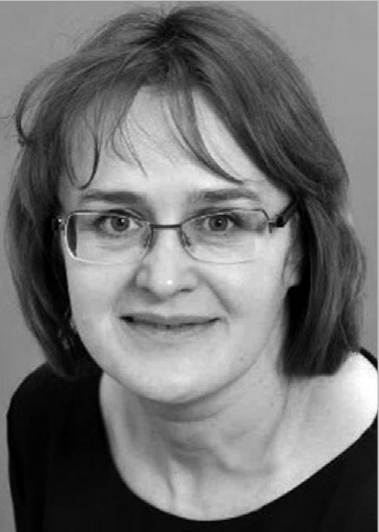

## Supporting information

As a service to our authors and readers, this journal provides supporting information supplied by the authors. Such materials are peer reviewed and may be re‐organized for online delivery, but are not copy‐edited or typeset. Technical support issues arising from supporting information (other than missing files) should be addressed to the authors.

Supplementary
