# Supplementary material for: Tin Oxide Based Nanomaterials and Their Application as Anodes in Lithium‐Ion Batteries and Beyond
Source: ChemSusChem. 2019 Aug 30;12(18):4140–59. doi: 10.1002/cssc.201901487 (PMC6790706; doi:10.1002/cssc.201901487)
Supplement: Supplementary file 1 — Supplementary [file CSSC-12-4140-s001.pdf]

Author Contributions

D.F. Writing - Review & Editing: Lead
F.Z. Writing - Original Draft: Equal
D.B. Writing - Original Draft: Equal
T.B. Writing - Review & Editing: Equal.
